# Serum Mass Spectrometry Proteomics and Protein Set Identification in Response to FOLFOX-4 in Drug-Resistant Ovarian Carcinoma

**DOI:** 10.3390/cancers15020412

**Published:** 2023-01-08

**Authors:** Domenico D’Arca, Leda Severi, Stefania Ferrari, Luca Dozza, Gaetano Marverti, Fulvio Magni, Clizia Chinello, Lisa Pagani, Lorenzo Tagliazucchi, Marco Villani, Gianluca d’Addese, Isabella Piga, Vincenza Conteduca, Lorena Rossi, Giorgia Gurioli, Ugo De Giorgi, Lorena Losi, Maria Paola Costi

**Affiliations:** 1 Department of Biomedical, Metabolic and Neural Sciences, University of Modena and Reggio Emilia, Via Campi 287, 41125 Modena, Italy; 2 Department Life Sciences, University of Modena and Reggio Emilia, Via Campi 103, 41125 Modena, Italy; 3 Seràgnoli Institute of Hematology, Department of Experimental, Diagnostic and Specialty Medicine, Bologna University School of Medicine, S. Orsola Malpighi Hospital, 40138 Bologna, Italy; 4 Department of Medicine and Surgery, Clinical Proteomics and Metabolomics Unit, University of Milano-Bicocca, 20126 Vedano al Lambro, Italy; 5 Clinical and Experimental Medicine (CEM) Doctorate School, University of Modena and Reggio Emilia, Via Campi 287, 41125 Modena, Italy; 6 Department of Physics, Informatics and Mathematics, Modena and Reggio Emilia University, Via Campi 213/A, 41125 Modena, Italy; 7 IRCCS Istituto Scientifico Romagnolo per lo Studio e la Cura dei Tumori (IRST), 47014 Meldola, Italy

**Keywords:** ovarian cancer, FOLFOX-4, mass spectrometry proteomics, serum samples, time lapse detection, network enrichment analysis, cancer molecular pathways, protein panel

## Abstract

**Simple Summary:**

A mass spectrometry (MS) proteomics and molecular pathway study was applied to serum samples of patients with ovarian serous carcinoma administered the FOLFOX-4 drug combination protocol, before the second cycle of therapy. This exploratory study aimed at identifying a protein panel that could be significantly modulated during two different collection time intervals and associated with patient response to therapy. The label-free differential MS proteomic analysis of 14 serum samples was conducted and identified 291 shared expressed proteins; 12 proteins resulted in being significantly associated with response to treatment and time of sample collection. The network enrichment analysis performed through STRING and other bioinformatic tools provided a metadata validation of the panel in which the identified proteins were related to resistant ovarian cancers at the molecular level. We concluded that the discovered protein panel that guided the identification of the associated molecular pathways could be further explored in a higher number of patients. Considering the lack of biomarkers that can guide the selection of further therapeutic approaches after drug resistance appearance, our study may suggest a new direction in the discovery and validation of a protein panel as biomarkers for future clinical application.

**Abstract:**

Ovarian cancer is a highly lethal gynecological malignancy. Drug resistance rapidly occurs, and different therapeutic approaches are needed. So far, no biomarkers have been discovered to predict early response to therapies in the case of multi-treated ovarian cancer patients. The aim of our investigation was to identify a protein panel and the molecular pathways involved in chemotherapy response through a combination of studying proteomics and network enrichment analysis by considering a subset of samples from a clinical setting. Differential mass spectrometry studies were performed on 14 serum samples from patients with heavily pretreated platinum-resistant ovarian cancer who received the FOLFOX-4 regimen as a salvage therapy. The serum was analyzed at baseline time (T0) before FOLFOX-4 treatment, and before the second cycle of treatment (T1), with the aim of understanding if it was possible, after a first treatment cycle, to detect significant proteome changes that could be associated with patients responses to therapy. A total of 291 shared expressed proteins was identified and 12 proteins were finally selected between patients who attained partial response or no-response to chemotherapy when both response to therapy and time dependence (T0, T1) were considered in the statistical analysis. The protein panel included APOL1, GSN, GFI1, LCATL, MNA, LYVE1, ROR1, SHBG, SOD3, TEC, VPS18, and ZNF573. Using a bioinformatics network enrichment approach and metanalysis study, relationships between serum and cellular proteins were identified. An analysis of protein networks was conducted and identified at least three biological processes with functional and therapeutic significance in ovarian cancer, including lipoproteins metabolic process, structural component modulation in relation to cellular apoptosis and autophagy, and cellular oxidative stress response. Five proteins were almost independent from the network (LYVE1, ROR1, TEC, GFI1, and ZNF573). All proteins were associated with response to drug-resistant ovarian cancer resistant and were mechanistically connected to the pathways associated with cancer arrest. These results can be the basis for extending a biomarker discovery process to a clinical trial, as an early predictive tool of chemo-response to FOLFOX-4 of heavily treated ovarian cancer patients and for supporting the oncologist to continue or to interrupt the therapy.

## 1. Introduction

Ovarian cancer is the most lethal gynecological malignancy, and despite the relatively high response rate to first-line standard chemotherapy, most patients develop recurrent disease [1]. Treatment is performed with carboplatin in combination with paclitaxel, and then other chemotherapeutic drugs are used such as topotecan and gemcitabine as second line therapy, as well as pegylated liposomal doxorubicin hydrochloride (PLDH), alone or alongside platinum drugs [2]. More recently, antiangiogenetic drugs or PARP inhibitors have entered into clinical trials and therapy [3]. Patients who progress or relapse within 6 months after the end of the treatment have been reported to have the worst outcomes [4]. Therefore, these patients often undergo multiple chemotherapy courses to try to achieve long-term remission and an acceptable quality of life. This approach raises the risk of cumulative toxicity and/or absence of response. For this reason, new, effective, and less toxic therapies for patients with recurrent and persistent disease are needed. Some in vitro studies have indicated a potential synergy between oxaliplatin and 5-fluorouracil (5-FU)/leucovorin (FOLFOX-4) [5,6]. While oxaliplatin is an alkylating agent, 5-FU is the prodrug of the widely used inhibitor of thymidylate synthase (TS), namely 5-fluoro-deoxyuridine-5′-monophosphate, (FdUMP) and it is incorporated in mRNA, thus, affecting DNA and protein synthesis [7]. Leucovorin, i.e., folinic acid, has been reported to favor TS inhibition and subsequent DNA-directed effects, because this folate analog is readily converted into 5,10-methylenetetrahydrofolate, the TS cofactor needed for ternary-complex formation [8,9]. This combination represents a standard regimen in the management of some advanced tumors such as colorectal (CRC), gastric, and breast cancers [10,11,12,13], and more recently, as salvage treatment in refractory or resistant ovarian cancer [14]. 

**Figure 1:**
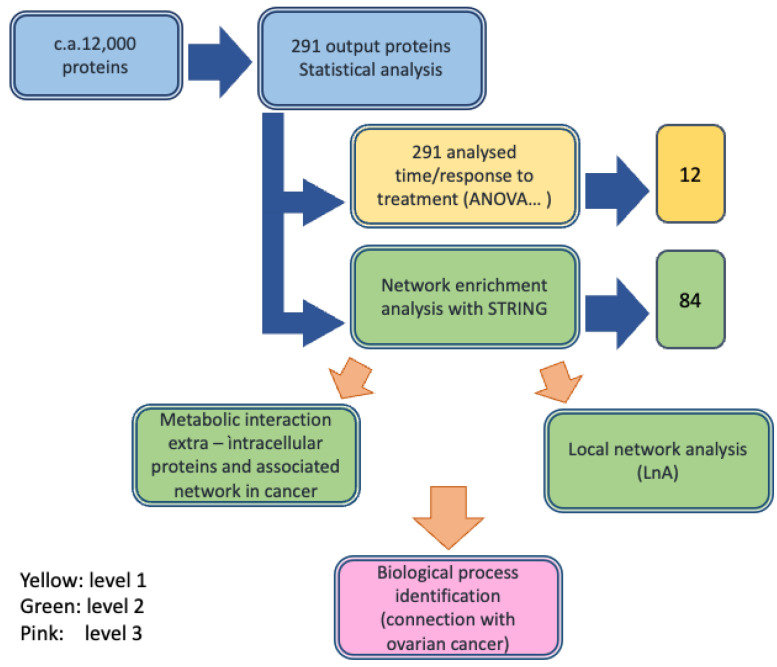
Workflow of the protein identification process in ovarian cancer serum samples performed through label free differential mass spectrometry analysis and integration with the bioinformatic analysis.

Circulating biomarkers detected in the serum of ovarian cancer patients are involved in either the cause of a malignancy or a systemic response to a malignancy. These factors may originate from several sources including the tumor itself, the surrounding stroma, or systemic tissues involved in the host response. It is unclear how circulating biomolecules are biochemically related to their respective producing sources and how their expression change is associated with cancers. In general, the overlap between the expression of proteins in cancer cells and in serum is limited. In the case of ovarian cancer, a tissue-derived effect in serum has been suggested [15]. Investigations of chemotherapy mechanisms and the discovery of the factors responsible for chemotherapy response and resistance may provide a selection of alternative therapeutic options or chemo-sensitizing agents [16,17]. Several techniques, including MALDI-TOF MS, ESI-TOF MS, and associated bioinformatics analysis, have been proposed to investigate proteome changes during cancer treatment [18,19,20,21,22,23,24]. Almost all the available studies have been performed on samples collected before treatments, and only a few investigations have been run on samples collected and analyzed sometime after treatment and compared with data before treatment to correlate the changes in protein levels with patients responses [18,19]. We wonder if we could apply a similar approach to a difficult case, such as in serum samples derived from heavily pretreated ovarian serous carcinomas patients observed in a routine clinical practice [14]. In this study, the chosen patients were resistant to platinum drugs and had undergone chemotherapy with different drug combinations, and then the FOLFOX-4 regimen as a salvage therapy. According to our methodology, we started with an experimental approach for a serum sample MS analysis by considering the differentially expressed proteins (DEP) between T0 and T1 in two groups of patients, i.e., nonresponders (NR) and partial responders (PR), and an association of the protein changes with patients’ responses was found. Finally, we compared the experimental results with the results of a metadata analysis, which supported the biological significance of the experimental finding. We considered that the protein panel identified was a useful suggestion for performing a biomarker discovery on a larger number of patients. The overall approach is described in Figure 1.

## 2. Materials and Methods

### 2.1. Experimental Design

The current study is a retrospective translational analysis of serum samples from patients who received FOLFOX-4 as salvage chemotherapy for the treatment of recurrent ovarian carcinoma (OC). The medical scientific committee of the IRST Istituto Scientifico Romagnolo per lo Studio e la Cura dei Tumori approved the study (protocol # 5108/V.3) [14]. All the patients shared the following characteristics from baseline serum collection (T0) to T1 collection: (i) no progression of the disease after the first cycle of FOLFOX-4 administration (T1); (ii) no adverse effect reported after T1; (iii) distinction between PR and NR was assessed according to the guidelines [25,26], with a follow-up evaluation 6 months after the FOLFOX-4 cycle. The details are reported in Supplementary Materials S1 and Table S1. The MS translational study consisted of collecting two serum samples from the patients, in which the baseline sample was before treatment at T0 and the second sample was before the second cycle of treatment (T1). We selected 7 patients who shared a similar pharmacological treatment and, overall, evaluated 14 serum samples. According to our methodology, we started with the experimental approach to serum sample MS analysis, and then we statistically reduced the number of proteins that resulted in being significantly differentially expressed to a panel of 12 proteins. On the resulting protein panel, we performed a bioinformatic analysis through network enrichment proteomic tools (see Section 2.5 below). 

### 2.2. Serum Immunodepletion of Albumin and IgG’s

A ProteoPrep Immunoaffinity Albumin and IgG Depletion Kit (Sigma-Aldrich) was employed to remove albumin and IgG from the collected serum samples and to enrich the samples with low abundant proteins (LAPs). First, the storage solution was removed, and the columns were equilibrated, as reported by the vendor. Then, 50 µL of serum samples were diluted with equilibration buffer and loaded onto the wet columns with 10 min incubation to promote binding between albumin/IgG and the resin. 

To maximize the recovery of the unbound protein fractions (i.e., LAPs), the columns were washed with 125 µL of equilibration buffer and centrifuged for 60 s. The obtained aliquot contained the majority (>95%) of the LAPs (checked by SDS-page). To collect the remaining trace amounts of unbound proteins from the column, 0.4 mL of ProteoPrep immunoaffinity equilibration buffer were added and centrifuged at 8000 × *g* for 60 s. The obtained LAP fractions were quantified with a Bradford assay on 96-wells plate. 

### 2.3. Protein Digestion via Filter-Aided Sample Preparation (FASP)

First, 0.2 mg of LAPs were incubated with 20 µL of 0.1 M dithiothreitol (DTT, Sigma-Aldrich, St Louis, MO, USA), 0.2% Protease Max trypsin enhancer (Promega, Madison, WI, USA) in 50 mM ammonium bicarbonate (ABC, Sigma-Aldrich) on Microcon YM-30 (Millipore, Burlington, MA, USA) filters at 55 °C for 30 min, and centrifuged at 14,000× *g* for 15 min. Then, 200 µL of UA buffer (8 M urea in 0.1 M Tris/HCl, pH 8.5) and 100 µL of 50 µM iodoacetamide IAA (Sigma-Aldrich, St Louis, MO, USA) were added, in two steps, to reduce disulfides and alkylate cysteines, followed by centrifugation at 14,000× *g* for 15 min. The samples were digested overnight with 4 µL of 1 mg/mL trypsin solution (Promega Corporation, Madison, WI, USA) in ABC at 37 °C, 250 rpm. 

After adding 40 µL ABC, FASP filters were centrifuged again at 14,000× *g* for 30 min to collect hydrolyzed peptides. Finally, the samples were acidified with CF_3_COOH, desalted with C18 SPE cartridge (7 mm × 3 mL, 3M Empore, Maplewood, MN, USA), and dried down (Speedvac, Eppendorf, Hamburg, Germany). The samples were stored at −80 °C until MS analysis.

### 2.4. LC-MS/MS Label-Free Quantification

The MS analysis was performed with an nLC coupled online with Impact HD™ URH-TOF (Bruker Daltonics GmbH, Bemen, Germany). The samples were resuspended in mobile phase, quantified by NanoDrop assay, and analyzed using an ultra-high resolution (UHR) Impact HD^TM^ QTOF mass spectrometer, at the University of Milano Bicocca, Monza. Each sample was injected three times and separated with a Dionex nRSLC (Rapid Separation LC nano, ThermoScientific, Sunnyvale, California, USA) before on-line desalting. A multistep gradient ranging from 4 to 98% acetonitrile in 240 min at a flow rate of 300 nL/min was applied [24]. Peptides were analyzed in data-dependent acquisition (DDA) mode and both an internal mass calibration segment (Na Formate, 15 min length, before the beginning of the gradient and the injection of the actual samples) and a 1221.9906 m/z lock mass, during the proper sample analysis, were employed in each run to retain mass accuracy.

Raw spectral data from DataAnalysisTM v.4.0 Sp4 (Bruker Daltonics, Germany, GmbH, Bemen, Germany) were processed with the Mascot search engine, through Mascot Daemon. Database matching was restricted to human SwissProt (October 2019, 561,356 sequences and 201,858,328 residues) [27]. The searching parameters were set as follows: trypsin as enzyme, carbamidomethyl (C) as fixed modifications, oxidation (M) as variable modification, 20 ppm mass tolerance for MS^1^ and 0.05 Da for fragments. An automatic decoy database search for FDR calculation and a built-in percolator algorithm for rescoring peptide-spectrum matches were applied [28]. The Progenesis QI for proteomics v. 2.0 (Nonlinear Dynamics, Newcastle, England) platform was used for label-free data elaboration and the determination of the normalized relative abundances of identified proteins and peptides [29]. Briefly, the alignment process was conducted based on the ion intensity maps of all imported runs. To compensate for between-run variation in LC separation, and to maximize the overlay across the data, alignment scores above 60% were accepted per each run. All matched proteins were used for total ion current (TIC) normalization. The Mascot software search engine was used for protein identification, setting the “search parameters” software option as described above. Only non-conflicting peptides were selected for determining the fold change to prevent the overlapping of trends derived from different proteins that share the same peptides. 

The results were validated with an UHPLC coupled to an Orbitrap Q-Exactive equipped with a micro-ESI (Thermo Fisher Scientific, Waltham, MA, USA). Two samples (F10 and F12) at T0 were processed as described in Section 2.3 and Section 2.4 and analyzed in replicate. Inclusion lists of 3–4 unique peptides per each DEP were generated with PeptideAtlas [30] and included in a target list of the software (XCalibur, Thermo Fisher). Peptides were separated with 180 min gradient in a C18 Hypersil Gold column, 100 × 2.1 mm, 1.9 µm column (Thermo Fisher Scientific, Waltham, MA, USA) in ddMS^2^ acquisition mode (Top8). Peak lists were analyzed with Mascot Matrix for protein matching and Progenesis QI Proteomics (Nonlinear Dynamics, Newcastle, UK) [31,32]. One-way ANOVA was employed to perform the differential analysis. The MS parameters and statistical analysis of peptides/proteins used for the semitargeted validation experiment are described in Supplementary Materials, Tables S2 and S3.

### 2.5. Proteins Network Analysis

The STRING (www.string.org) [33] (accessed on 2 October 2021) protein–protein interaction networks functional enrichment analysis was performed as follows: the input proteins were the 12 selected proteins plus thymidylate synthase (TYMS) and dihydrofolate reductase (DHFR). The addition of TYMS and DHFR is justified by the fact that 5-FU nucleotide targets TYMS and the TS cycle, and therefore, also affects DHFR concentrations. Leucovorin counteracts DHFR activity of the dihydrofolic acid reduction effect. The analysis settings adopted were the following: multiple proteins mode; interaction score in the range 0.400–0.700 (medium to high confidence), depending on the experiment’s objectives; all selection criteria checked, excluding neighborhood; 1st shell max 10 interactions; 2nd shell max 50 interactions; confidence mode represented by the edge thickness related to the strength of the confidence (the highest, the larger). Other specific parameters included: number of nodes, 73; number of edges, 409; average node degree, 9.86; average local clustering coefficient, 0.663: expected number of edges, 174; PPI enrichment *p*-value <1.0 × 10^−16^. We observed that by changing the parameter settings, the global network did not change regarding the main biological pathways present during the STRING enrichment process. The SOD-GPX redox biological process was always present together with the vesicles and trafficking system and the lipoprotein network. 

#### 2.5.1. Proteins Localization

Gene localization was analyzed according to UniProtKB [34] and the Compartment Subcellular localization database, as well as cellular component ontologies visualized by the Gene Ontology (GO) Consortium (GeneCards, https://www.genecards.org, accessed on 2 October 2021) [35]. Subcellular localizations from compartment localization data were integrated from the literature manual curation, high-throughput microscopy-based screens, predictions from primary sequence, and automatic text mining [36]. Unified confidence scores of the localization evidence were assigned based on evidence type and source and ranged from 1 (low confidence) to 5 (high confidence) [37]. The assumption was made that the proteins in the extracellular space could be present in the serum [38]. Original content and additional information can be found at the Human Protein Atlas available at www.proteinatlas.org [39] and GeneCards [35]. 

#### 2.5.2. Local Networks

Local network interactions generated for each of the 12 proteins of the panel were obtained through STRING upon elaboration of data from GeneCards databases, Reactome (https://reactome.org, accessed on 2 October 2021) [39], and Panther (http://www.pantherdb.org, accessed on 2 October 2021) [40]. For each protein, the minimal number of interactions were considered that could explain the connections among the serum protein, the membrane protein, and at least one intracellular protein. Relevant interconnections are based on a confidence score >0.700 and *p*-value <1 × 10^−16^. 

### 2.6. Statistical Methods

The sample size was chosen according to the availability of heavily treated OC patients who agreed to participate in the study. Despite the low numerosity of the sample to statistically validate a single biomarker (i.e., this study is intended to provide only a first step into drug resistance circulating markers); the statistical approach adopted during MS analysis and metadata integration have been shown to provide robust outcomes [21]. All the serum samples were treated in duplicate and analyzed in triplicate in LC-MS to eliminate both biochemical and instrumental biases. Statistical analyses were conducted using R language and environment for statistical computing (R Foundation for Statistical Computing, Vienna, Austria). A type I error of 5% was taken as the limit for two-sided *p*-value statistical significance and all confidence intervals (CI) were reported as 95% CI. Differential protein abundance intended as fold change (FC) over time between patient response stratification was analyzed by mean of a paired *t*-test. A volcano plot was adopted to show the results of the FC analysis and to highlight which proteins had a statistically significant behavior change. Response to treatment contribution over time and its interaction with the different timepoints was investigated by mean of mixed-effect analysis of variance (ANOVA).

## 3. Results

### 3.1. Study Design

First, we developed the MS study to identify the detectable proteins, their characterization, and their chemical properties such as MW and abundance. Then, statistical analyses were performed to identify the DEPs and selection criteria were applied to pass from about 12,000 proteins detected overall during the MS experiments (observations) down to 291. After subsequent selection steps that included time response (T0–T1) and PR or NR group response, 12 proteins (protein panel) were finally identified and their biological roles were described in the context of cancers/ovarian cancer pathology. Then, their profiles were studied in terms of time and response and reported in an expression profile representation. The second level of investigation was based on bioinformatic analysis of the metabolic network of the 12 identified proteins to characterize their interconnections in human cells and their localizations in cells, on cellular membranes and outside cells, by database analysis (STRING, GeneCards, Reactome and Panther). The process, first, allowed the description of a global network, then a local network around each protein of the panel was identified, and their intra-/extracellular localizations were characterized. The third level was the annotation of the biological role of each of the selected proteins in the cell response to FOLFOX-4, through metadata analysis and their connection with ovarian cancer. The workflow design is reported in Figure 1.

### 3.2. Protein Identification and Characterization (First Analysis Level)

#### 3.2.1. Label-Free MS Proteomic Approach

**Figure 2:**
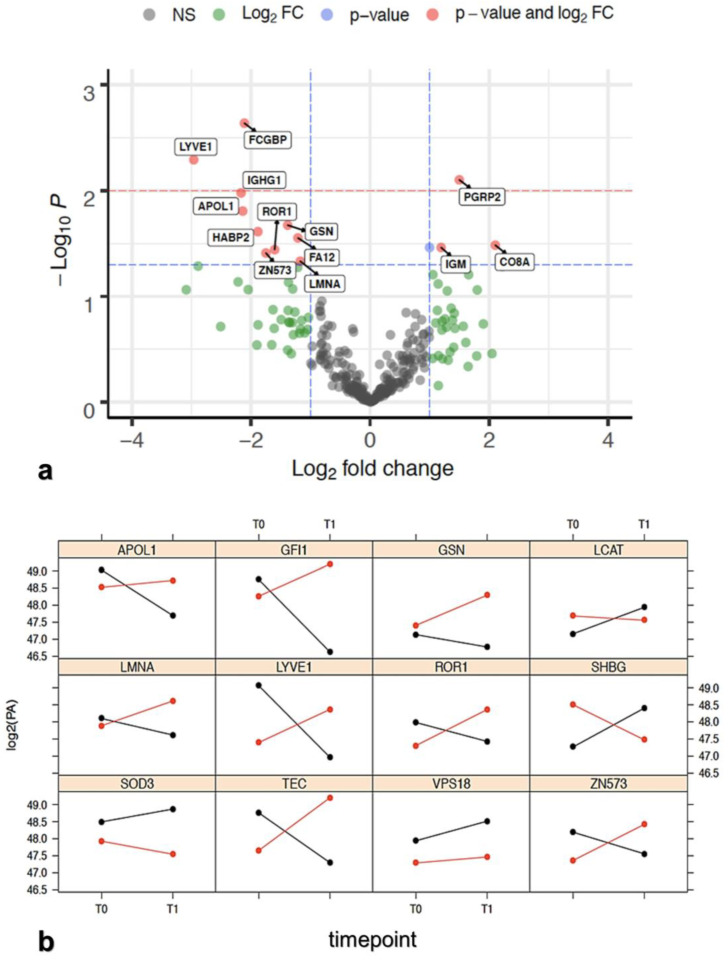
(**a**) Volcano plot showing -log_10_(*p*-value) versus log_2_(FC_R_/FC_NR_). Horizontal lines indicate 0.05 (blue) and 0.01 (red) *p*-values. Proteins statistically significant (*p* < 0.05) and with a FC > 1 were reported alongside with their names. Protein in red dot fits the FC and statistical significance criteria, blue dot fits only the statistical criteria, the green dot fits only the FC criteria, and the grey dot does not fit either criteria. Proteins over the blue dashed line showing *p* < 0.05 are reported in Table 1. Data for each protein were taken from the protein identification table of the MS analysis elaboration. (Supplementary Materials doi.org/10.15490/fairdomhub.1.datafile.4074.1); (**b**) log_2_ protein abundance expression profile between T0 and T1 relative to the 10 proteins reported in Table 2. SOD3 and VPS18, taken from Table S5a, are added as an example of non-intersecting proteins. The red color is related to NR patients, while the black color is related to PR. When the red and black lines intersect, it is intended that the contribution of the time in response status is relevant and, consequently, the interaction between response and treatment as well. Data are elaborated from the file named “Report progenesis _all rows_all data for biostatistics.doi.org/10.15490/fairdomhub.1. datafile.4074.1.”

Normalized protein abundance was the main parameter on which we analyzed 291 DEPs detected over two serum samples collected at T0 and T1 for each of seven patients (total 14 samples analyzed) and processed in three technical replicates, which resulted in a total of 12,222 observations. Then, these proteins were analyzed to identify DEPs between response groups. Firstly, we examined the differential protein abundance as fold change (FC) over time between PR and NR and a paired *t*-test was applied considering an alfa error <0.05 for statistical significance. The results from this analysis were reported by means of a volcano plot (Figure 2a), and only a small subset of proteins (13/291) showed reproducible statistically significant FC between the PR group versus NR group. The LCAT protein, in Figure 2a, has no label because its FC is under lower threshold (FC = 0.9982). LCAT (phosphatidylcholine-sterol acyltransferase) is indicated with the blue dot, which exceeds the limit of *p*-value, but not that of FC. Ten out of fourteen DEPs showed a reduction of log_2_FC(T1/T0) in the PR group with respect to NR, confirming that downregulated proteins outnumbered upregulated proteins (Table 1, right column). As expected, a confirmation of DEPs identified (Table 1) that reported a statistically significant differential expression between PR and NR groups at time T0 and T1 was obtained also with the ANOVA test (Table 2).

To confirm the identified protein list reported in Table 2, we applied a semitargeted approach in which the proteotypic peptides of samples F10 and F12 at T0 were analyzed (Supplementary Materials S2 and Table S2, Table S3). The samples were selected because they showed the largest differences in the protein profiles (raw AUC quantification of non-conflicting peptides), as represented in the raw data report (doi.org/10.15490/fairdomhub.1.datafile.4074.1). The sample analysis revealed the presence of the 12 proteins selected from this study (Table 2). Data elaboration was robust and consistent with the primary label-free MS investigation reported in the manuscript. The results suggest that single proteotypic peptides in the LC-MS/MS inclusion list can be used as a means to estimate the abundance of the corresponding entire protein in serum samples (semitargeted approach). The description of the work is reported in the in the raw data report (doi.org/10.15490/fairdomhub.1.datafile.4074.1) associated with this manuscript (see data availability statement).

#### 3.2.2. Protein Selection at T0–T1 Timepoints

Initially, we proceeded by statistically analyzing the DEP by means of a linear mixed-effect analysis of variance (ANOVA) that allowed us to evaluate the independent contribution of time (T0–T1, timepoint) and response or their iterative effect on protein expression (Supplementary Materials, Tables S4 and S5). Protein abundance in log_2_ scale was chosen as the dependent variable, while time and response to therapy were included as independent covariates with a fixed effect. Lastly, the same study was conducted considering a random effect. The results were interpreted as statistically significant when beta error was ≤0.20 and alfa error was <0.05.

Response to treatment has a statistically significant contribution on differential expression of five proteins (Table S5a), otherwise protein differential expression of a different set of 27 proteins was identified as mainly driven independently by timepoint (Table S5b). Lastly, 20 proteins demonstrated an iterative effect of treatment response and timepoint on their differential expression (Table S5c). Table S5a shows proteins that are different from those present in Table S5c. If we consider the baseline sample, the proteins associated with patient outcome, and therefore, those which are potentially able to predict which patients can respond to therapy or not, are different from those suggesting that the patients are responding to therapy after the beginning of the same. Thus, the two protein panels have a different meaning, with the latter being of more clinical interest. A time-dependent ANOVA between NR and PR samples (T0 vs. T1 timelapses) evidenced a list of significant proteins (Table S5c), which was submitted to further analysis. 

For the composition of the final panel, we progressed with the selection based on the biological significance of the 20 proteins and their clinical relevance in cancer processes. Then, during the network enrichment analysis process we added two proteins, SODE (SOD3) and VPS18, as they were differentially expressed based on response to treatment (Table S5a), and they played important roles in cellular pathways related to cancer protection from reactive oxygen intermediates (SOD3) and autophagy (VPS18), respectively, (Table 3) [41].

#### 3.2.3. Role of the Selected Proteins in Cancer Processes

A metadata investigation was performed to validate the proteins in the panel. The up- or downregulation trend for each of the 12 proteins of the final panel at T0 and T1, is reported in Figure 2b. These trends are statistically significant and specifically influenced by one or both variables (time and response) or by their interaction, which is the specific case when they intersect. An intersection of the trends indicates that one of the two variables influences the state of the other. In our case, the state of time (T0 and T1) influences the state of the answer. Indeed, the group that had an overexpression at T0 decreased at T1, and vice versa. Only GSN, SOD3, and VPS18 proteins over 12 did not intersect. The biological properties of the 12 proteins of the panel are summarized in Table 3 and Table S6. The biological roles of the 12 proteins and the variations of their expression with respect to patient outcome and their roles in ovarian cancer are given below. 

The lymphatic vessel endothelial hyaluronan receptor-1 (LYVE-1) is a hyaluronic acid receptor, which is selectively expressed in the endothelium of lymphatic capillaries [42]. Serum low LYVE-1 levels have been significantly associated with the occurrence of distant metastases in some cancers [43]. LCAT and sex hormone-binding globulin (SHBG) have been recorded as differentially expressed between PR and NR patients. SHBG is present in serum and plasma (GeneCards). Although SHBG was not associated with overall risk of ovarian cancer in one recent study [44], both LCAT and SHBG downregulation have been reported to provide important information on the aggressiveness of the ovarian cancer [45]. This trend was also observed in our studies (Figure 2b), where both proteins decreased in NR and increased in PR. It is worth noting that deregulated lamin-A/C (LMNA) expression has been associated with nuclear shape, mechanical stability, and migration ability of cells in ovarian cancer [46,47]. In our experiments, LMNA increased between T0 and T1 in NR patients and decreased in PR patients (Figure 2b), therefore, the trend agreed with these studies. 

Two members of the tyrosine kinase family, non-receptor tyrosine-protein kinase Tec (TEC) and receptor tyrosine kinase-like orphan receptor 1 (ROR1), were found differentially expressed in PR with respect to NR, in baseline samples. TEC kinase, together with other proteins, play a role in the intracellular signaling of both B and T lymphocytes, relevant cells that contribute to the tumor microenvironment which is increasingly interested in controlling cancer growth [48]. ROR1 overexpression has been associated with a poor prognosis in several solid and hematological malignancies, including ovarian cancer [49,50] and other malignancies [51]. The same trend was also observed for ROR1 that showed higher expression at T1 in NR, while its expression was lower in PR (Figure 2b). 

We observed lower gelsolin (GSN) levels in sensitive (PR) patients as compared with their chemo-resistant counterparts (NR). GSN is considered to be one important determinant for chemo-resistance, probably due to inhibition of the apoptosis pathways [52,53]. SOD3 (extracellular superoxide dismutase [Cu-Zn]) is the predominant antioxidant enzyme secreted into the extracellular space, that affects drug delivery, and chemotherapeutic effect on tumors [41,54]. It was selected because it significantly correlated with response to treatment (Table S5b), but its level change was not correlated with time T0–T1. We observed higher SOD3 levels in PR patients with respect to NR patients in samples before treatment (Figure 2b), in agreement with the reported findings in the literature [54]. 

In our analysis, two zinc finger proteins were found to be differentially expressed between PR and NR patients: zinc finger protein (GFI1) and zinc finger protein 573 (ZNF573). In both cases, the two proteins demonstrated a significant statistical effect of treatment response and timepoint (Table S5c) on their differential expression. The protein encoded by ZNF573 decreased in PR, whereas increased in NR, at T1 time, suggesting a possible role of this protein in the response to treatment. The function of ZNF573 is still undetermined, and only a recent study has suggested that ZNF573 may be involved in cervical cancer activating the cancer progression through the regulation of transcription or structural integrity of cells [55].

We observed a similar trend for GFI1. Two recent studies reported that GFI1 has been shown to favor the survival of myeloid cells in myeloproliferative disease [56] and tumor maintenance in medulloblastoma [57].

Vacuolar protein sorting-associated protein 18 homolog (VPS18) was selected because it was significantly correlated with response to treatment (Table S5b). It was found to be overexpressed in PR with respect to NR in patients’ samples before treatment and was slightly increased between T0 and T1 in both cases (Figure 2b). It is well known that VPS18 plays a key role in vesicle-mediated protein trafficking to lysosome including the endocytic membrane transport and autophagic pathways [58,59].

The role of apolipoproteins in cancer has not been explored deeply yet. In our analysis, we found only apolipoprotein L1 (APOL1) differentially expressed between PR and NR patients. APOL1 is a secreted high-density lipoprotein, which binds to apolipoprotein A-I. It has been characterized as a novel Bcl-2 homology domain 3 (BH3)-only lipid binding protein [60,61]. In our studies, APOL1 decreased at T1 in PR with respect to NR (Figure 2b). Summarizing, the 12 proteins of the selected panel, considered to be relevant in the statistical analysis, are also confirmed to be relevant by metadata analysis. In fact, experiments in the literature have suggested that the proteins of the panel and their trends have also been similarly found in other ovarian cancer studies. In the next steps, we analyze how the 12 proteins are connected and which biological processes are involved using the enrichment network analysis approach. 

An RI cluster analysis with a zero inflated model was applied to the 12 DEPs to analyze their behaviors both at T0 only and with a differential model between T0 and T1. The most significant clusters identified were APOL1 + GFI1 + LYVE1 (zI = 4.20) and LCAT + LMNA (zI = 3.25) at baseline time only, whereas the cluster GFI1 + LCAT + LMNA was identified with a T0–T1 analysis with a zI = 3.67. If a Q-test was applied to discard the outlier data from proteomic triplicates (confidence interval >95%), the cluster APOL1 + GFI1 + LYVE1 emerged with a zI = 3.16. The results of the analysis are reported in Supplementary Material S7 and Table S7. 

### 3.3. Enrichment of the Cellular Network of the Selected Protein Panel (Second Analysis Level)

#### Global Network Analysis

The serum proteins relate to membrane proteins, and intracellular proteins are connected to each other through intracellular physical and functional networks. Therefore, it is relevant to identify the meaning of these connections. As a starting point, we characterized the panel of 12 proteins considering the overall metabolic network independently on the proteins’ localization, (cytosol, membrane, or serum) applying a global network analysis through STRING and GeneCards. This approach allowed the extraction of the necessary information for further explanation of metabolic changes in response to treatment. 

The first level of enrichment was the addition of thymidylate synthase (TYMS gene encoding for TS protein) and dihydrofolate reductase (DHFR). TS and DHFR represent the main proteins of the TS cycle, and therefore, it is expected that 5-FU (FOLFOX-4) modulates both [9,10,62]. Our MS experimental conditions did not allow identification of either TS and DHFR, as they are difficult to detect in differential proteomic experiments on tissue cancer samples due to their nuclear compartmentalization and low physiological concentrations, despite their recognized relevant role in cancer and drug resistance [62,63]. TYMS has also been considered a potential prognostic biomarker of overall survival in patients with CRC, where high TYMS levels predict for low overall survival [10].

The 12 selected proteins plus TS and DHFR were processed using STRING with their annotation to highlight any common biological processes in which they are involved and to identify their interconnections (Figure S1).

The UniProt entry names were used for the statistical over-representation test in STRING [64]. An enrichment analysis was performed and resulted in up to 84 total proteins divided in the first shell (12 proteins submitted plus TS and DHFR, colored spheres in Figure S1) and 69 extra proteins almost all belonging to the second shell (white color in Figure S1). Despite different attempts to connect all the 12 proteins plus TS and DHFR of the panel during the enrichment process, a few of them remained unconnected, specifically, GFI1 and ZNF573, at the level of enrichment selected. Three additional proteins, ROR1, LYVE, and TEC, showed a very limited connection with only one protein of the global network. The other proteins very well interconnected.

**Figure 3:**
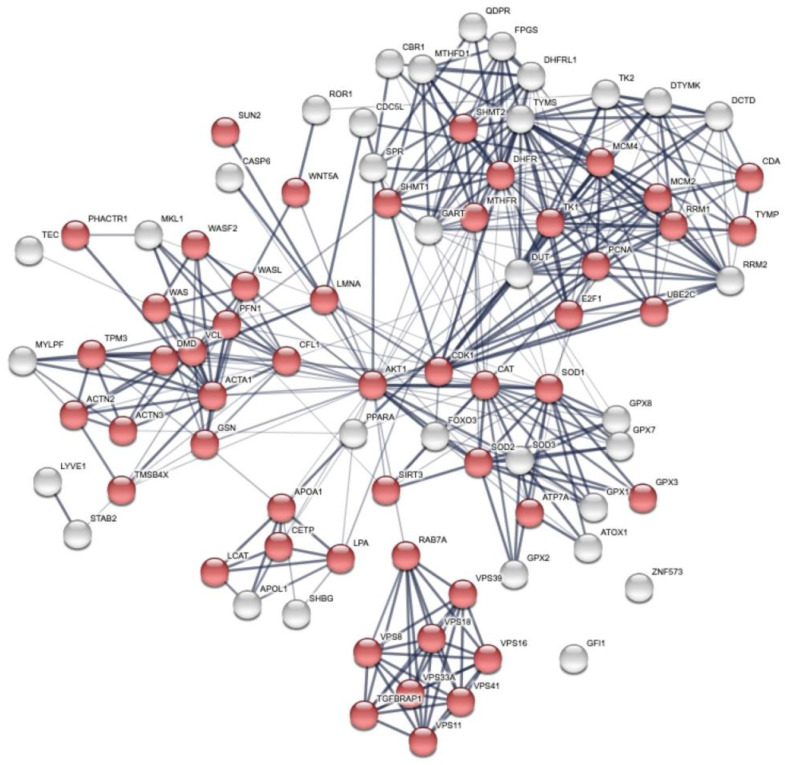
Global network visualization based on STRING pathway enrichment analysis of the 12 DEP proteins + TYMS and DHFR showing the most extended biological process containing the protein panel. Details are reported in the main text. Red spheres represent the cellular metabolism organization biological process with the following STRING features: GO:0016043 and FDR 51/5163.

Then, we studied the biological processes using the gene ontology (GO) [37] analysis of the pathways and biological processes and revealed that modulation of the cellular metabolism by FOLFOX-4 results from the combination of multiple layers of regulation. The overall network is dominated by the cellular organization biological process (Figure 3, red spheres).

**Figure 4:**
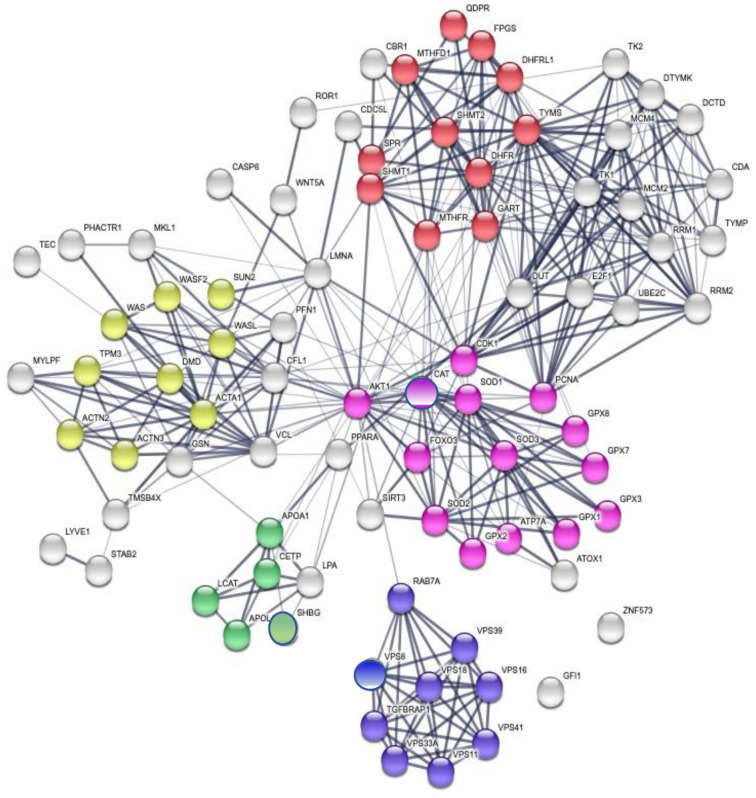
Global network visualization based on the STRING pathway enrichment analysis of the 14 selected proteins (12 + TYMS or TS and DHFR). The network shows the most relevant biological process containing the protein panel. Details are reported in the main text. The STRING features are the following: yellow spheres, actin-filament based movement and regulation (GO:0030048, FDR 1.31 × 10^−7^, 9/105); green spheres, cholesterol metabolic process (GO:FDR 2.3 × 10^−3^); violet spheres, endosome to lysosome transport (GO:0008333, FDR 1.78 × 10^−8^, 8/49); pink spheres, cellular response to oxidative stress (GO:0034599, FDR 5.36 × 10^−10^, 14/22); red spheres, pteridine-containing compounds biological process (GO:0042558, FDR 9.37 × 10^−14^, 11/33). A detailed GO analysis shows that the four most relevant biological processes involving the protein panel are related to vesicle trafficking process, lipoproteins associated metabolic process, structural component modulation in relation to cellular apoptosis and autophagy, and cellular oxidative stress response. The principal biological processes are well connected to the purine metabolism and apoptotic process generated by STRING around the 5-FU and leucovorin targets, i.e., TYMS and the TS cycle protein, DHFR.

A detailed GO analysis showed that the four most relevant biological processes involving the protein panel are related to vesicle trafficking process, lipoproteins associated metabolic process, structural component modulation in relation to cellular apoptosis and autophagy, and cellular oxidative stress response (Figure 4). These principal biological processes were well connected to the purine metabolism and apoptotic process generated by STRING around the 5-FU and leucovorin targets, i.e., TYMS and the TS cycle protein, DHFR.

Our analysis was based on protein modulations detected in the serum samples and related to intracellular biological processes. With the aim to understand the interconnections between serum and intracellular networks and how this is rationally associated with cancer biology, we conducted a local network analysis (LnA), and then a localization characterization of the proteins of the panel was performed, stemming from the global network analysis of Figure 4.

**Figure 5:**
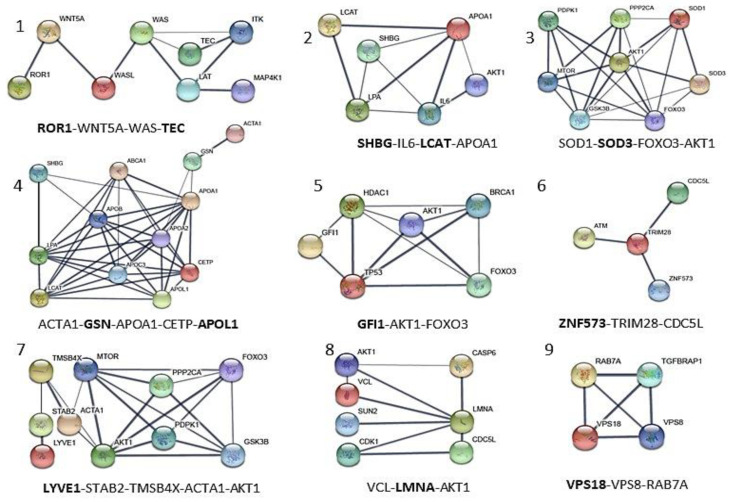
Local network interaction generated for each of the 12 proteins of the panel obtained through STRING upon elaboration of data from Figure 3 and GeneCards databases. The most relevant interconnections based on confidence feature (value of confidence >0.700 and *p* < 1.0 × 10^−16^) are also visualized in Figure 6. Each local network (1–9) shows the relevant protein connections written in the bottom, and proteins of the MS panel are reported in bold. SOD3 and VPS18, taken from Table S5a, are added as internal control proteins.

LnA was performed through the identification of the minimal number of interactions that each protein of the panel (Table 3) could establish with other interconnected proteins previously identified in the global network. A total of nine networks reported in Figure 5 describe the serum-membrane-intracellular connections for all proteins of the panel. A few local networks (1, 2, and 4) involve more than one protein of the panel. The function of ZNF573 was not well known and its network (number 6) was identified using the co-expression STRING analysis, and then only a few proteins were found connected through co-expression and experimental analysis to TRIM28 (tripartite motif containing 28, a protein coding gene), and then to CDC5L (cell division cycle 5-like, a DNA-binding protein involved in cell cycle control) (Figure 5). The detailed local specific protein-centered connections information is reported in Table S4. Some proteins are recurrently present in the networks such as AKT1, which is present in four of nine networks reported in Figure 5 (3, 5, 7, 8), while FOXO3, ACTA1, and APOA1 are present in two of nine networks. It is worth noting that AKT1, FOXO3, and APOA1 were not detected in the MS study, but are relevant in ovarian cancer development (Table S8). The local network identified, reported in Figure 5 are also found in the biological processes identified in the global network such as cholesterol metabolic process (LCAT and APOL1), actin-filament based movement and regulation (GSN, LMNA, and LCAT), endosome to lysosome transport and trafficking (VPS18), and cellular response to oxidative stress (SOD3) (Figure 4).

**Figure 6:**
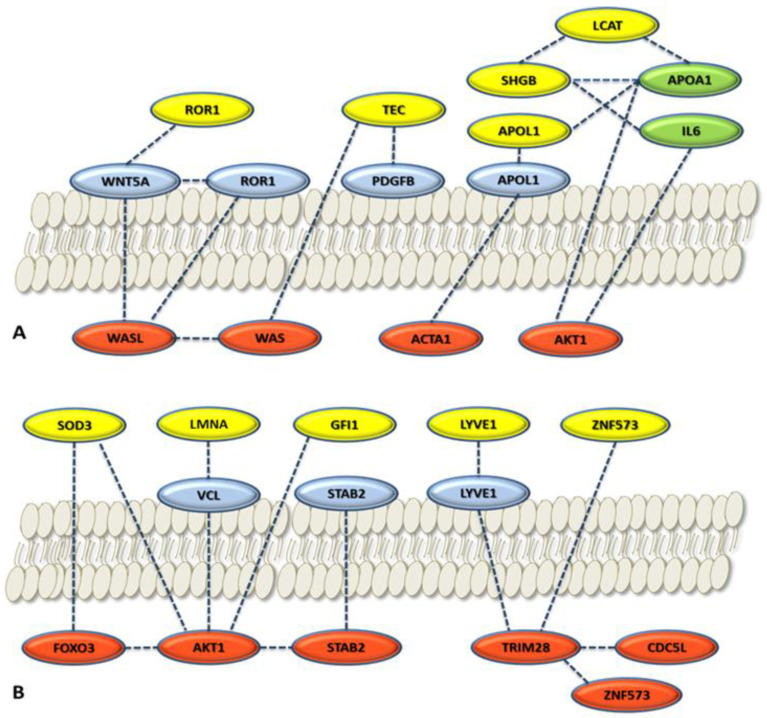
Local network connections for cytoplasmatic proteins (red), membrane proteins (blue), and serum proteins (yellow and green). The selected proteins of the panel were considered: (**A**) Panel proteins considered are ROR1, TEC, SHBG, LCAT, and APOL1; (**B**) panel proteins considered are SOD3, LMNA, GFI1, LYVE1, and ZNF573. Yellow circles, panel proteins selected; light green circles, relevant serum proteins for ovarian cancer in the network. Localization is based on GeneCards. The proteins reported not belonging to the panel come from the STRING local network analysis and GeneCards elaboration (Figure 5).

Localization of the proteins was achieved by GeneCards that adopted the Genome Atlas information [65]. Figure 6 shows the results of the localization network analysis. The protein connections are established between intracellular environment, plasmatic membrane, and extracellular space on the basis of metadata analysis through the different tools and database adopted. Some proteins are usually found in the serum such as SOD3, GSN (not shown), APOL1, LYVE1, and SHGB (Table 3). Some of them are related to their membrane protein form (ROR1, APOL1, and LYVE1) or to different proteins connected with the local and global networks. The localization metadata agree with the features of the protein of the selected protein panel.

**Table 3:** References of biological properties and main local interaction description of the panel of 12 proteins selected from those differentially expressed proteins. The network numbers from Figure 5 are reported in brackets. The proteins of the panel are indicated in bold.

Protein Code (Uniprot)	Protein Function	References
**APOL1**	Apolipoprotein A-I. Local network: **APOL1**-(CETP-**LCAT**-LPA-APOA1); APOA1-**GSN**-ACTA1; ACTA1-**GSN**-APOA1-CEPT-**APOL1 (network 4).**	[66]
**GSN**	Gelsolin. Local network: ACTA1-**GSN**-APOA1-CETP-**APOL1 (network 4).**	[52,53]
**GFI1**	Zinc finger protein Gfi-1; Transcription repressor essential for hematopoiesis. Local network: (BRCA1-**AKT1**-TP53-**GFI1**-HDAC1); (HDAC1-**AKT1**-FOXO3-BRCA1); (FOXO3-TP53-**GFI1**) (**network 5**).	[56,57]
**LCAT**	Phosphatidylcholine-sterol acyltransferase. Local network: **LCAT**-LPA-APOA1-**SHBG**-IL6-AKT1; **SHGB**-IL6-**LCAT**-APOA1 (**network 2**).	[44,45]
**LMNA**	Prelamin-A/C. Local network: VCL-AKT1-**LMNA**-CDK1; VCL-LMNA-AKT1 (**network 8**).	[46,47]
**LYVE1**	Lymphatic vessel endothelial hyaluronic acid receptor 1. Local network: **LYVE1**-STAB2-TMSB4X-AKT1-MTOR-FOXO3; **LYVE1**-STAB2-TMSB4X-ACTA1-AKT1 (**network 7**).	[42,43]
**ROR1**	Inactive tyrosine-protein kinase transmembrane receptor ROR1. Local network: **ROR1**-WNT5A-WAS-**TEC (network 1).**	[49,50,51]
**SHBG**	Sex hormone-binding globulin. Local network: **SHBG**-IL6-**LCAT**-APOA1A1 (**network 2**).	[44,45]
**SOD3**	(SODE) Extracellular superoxide dismutase [Cu-Zn]. Local network: SOD1-**SOD3**- AKT1-MTOR; SOD1-**SOD3**-FOXO3-AKT1 (**network 3**)	[41,54]
**TEC**	Tyrosine-protein kinase Tec. Local network: **TEC**-ITK-LAT-WAS-MAP4K1; **ROR1**-WNT5A-WAS-**TEC** (**network 1**).	[48]
**VPS18**	Vacuolar protein sorting-associated protein 18 homolog. Local network: **VPS18**-VPS8-RAB-7A (**network 9**).	[58,59]
**ZNF573**	Zinc finger protein 573. Local network: **ZNF573**-TRIM28-CDC5L (**network 6**).	[55]

### 3.4. Interaction between the 5-Fluorouracil Targeting Pathways and the Protein Set Identified in Serum (Third Analysis Level)

**Figure 7:**
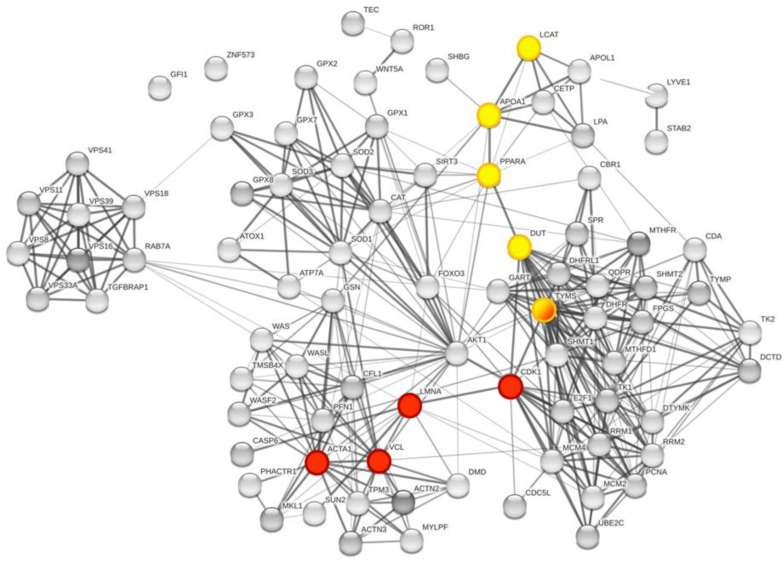
Global network visualization based on STRING pathway enrichment analysis focused on TYMS and DHFR. In the global network representation, the interaction pathway between the TYMS-DHFR protein network and the proteins of the panel identified through MS study are shown, respectively, in red and yellow. The represented nodes are the closest connection possible for the proteins of interest. The two main pathways identified are: TYMS-CDK1-**LMNA**-VCL-**ACTA1** (red nodes) and TYMS-DUT-PPARA-APOA1-**LCAT** (yellow nodes). TYMS is half yellow/half red.

We investigated how the TS protein target network and the associated replicative pathway targeted by 5-FU could modulate or interact with the selected serum protein panel by visual inspection of Figure 7. We followed the highest confident connections path, starting from TYMS, DHFR, and TK1, tyrosine kinases which represent the main proteins belonging to the pyrimidine synthesis, metabolism, and replication processes. These proteins can interact with key proteins such as deoxyuridine triphosphatase (DUT) and cyclin dependent kinase 1 (CDK1) that bridge the connections with other proteins of the serum panel. The two main pathways identified are: TYMS-CDK1-**LMNA**-VCL-**ACTA1** (red nodes in Figure 7) and TYMS-DUT-PPARA-APOA1-**LCAT** (yellow nodes in Figure 7). Other connections, however, are possible. The connections are supported by high confidence (connection edges are 0.9), the pathways are short (small number of proteins involved) and with at least two relevant nodes (colored spheres). It is worth noting that, in Figure 5, LMNA is present in local network 8, ACTA1 is present in networks 4 and 7, while LCAT is present in networks 2 and 4. AKT1 is present in networks 7 and 8 of Figure 5, and represents one relevant connector between the protein panel as a whole and 5-FU related proteins, as showed in Figure 6, where it is shown at the center of the global network, as a highly interconnected node.

## 4. Discussion

Over 12,000 proteins were detected in 14 serum samples, and a number reduction was required to select a protein panel that could be significantly associated with patient response to treatment and with the timepoint modulation. A valuable statistical analysis was usefully applied, and we obtained 12 proteins that were properly evaluated using a metadata analysis approach to understand their biological significance in ovarian cancer. We were able to link each of the 12 proteins to response to treatment. One specific feature of our study was the timepoint of sample collection, ranging from T0 (baseline sample, before treatment) to T1 (collected before the second cycle of treatment). From three to four weeks after treatment, a change of the proteom in cancer cells is expected, this change can be consistent with an early response to therapy, or no response [67,68,69]. 

To have a broader view of the overall connection pathways, first, we performed a network enrichment analysis including the 12 proteins of the panel with the additional FOLFOX-4 drugs target (TYMS and the TS cycle protein DHFR) as a part of a large metabolic network, without considering protein localization. The second step was to analyze each protein of the panel and its local network and set out whether each protein could be interconnected with the intracellular and membrane proteins in a signal transduction pathway. The global network analysis using the STRING system also included TYMS and DHFR as the major targets of 5-FU-derived drug and leucovorin, respectively (FOLFOX-4). The protein–protein interaction study showed that those proteins could modulate, at least in part, the serum proteins belonging to the panel identified through the proteomic study. 5-FU, by targeting TYMS and by decreasing its catalytic activity and protein levels, showed an impact on the protein functions of its own network (other folate dependent proteins) and was able to modulate LMNA and ACTA1 or LCAT. In this case, we consider that 5-FU directly modulates the cellular protein targets that are connected with those serum proteins we have identified (LMNA and LCAT) (Figure 5) and ACTA1 cellular protein. It is interesting to observe how, through the three above-mentioned proteins, many other proteins of the panel can be connected. The metadata analysis on the 12 proteins of the panel are consistent with the reported trend of each protein in cancer and specifically ovarian cancer. This trend was consistent with the literature findings for all proteins identified in the MS study, supporting the possible link between tumor tissue functions and the circulating serum proteins [43,45,47,52,54,56,70,71,72].

Next, we cross-examined the networks of protein–protein interactions to highlight the cancer-associated biological processes and the involvement with the treatment response. ROR1 was connected to Wnt5a. ROR1 activates the ROR1/Akt/p65 pathway, which is involved in inflammation and immune system [73] (Figure 5). Wnt5a overexpression has been implicated in the aggressiveness of diverse tumor types and has been shown to promote cell invasion and metastasis. Recently, several studies have shown that non-canonical Wnt signaling, such as canonical Wnt signaling, could induce cancer multidrug resistance (MDR) in several cancers, although with distinct mechanisms [70]. Moreover, ovarian cancer cell lines, characterized by high levels of Wnt5a expression, have shown lower sensitivity to several drugs (paclitaxel, oxaliplatin, 5-FU, epirubicin, and etoposide) [74,75]. It is worth noting that ROR1, in our study, was found to be downregulated in PR patients with respect to NR patients during the treatment (Table 2 and Figure 2b), in agreement with the biological rationale. It is noteworthy that AKT1 can degrade prelamin A (LMNA) [46], and that LMNA is involved in ovarian cancer [47]. In accordance with the above results, we found a decrease in LMNA in our panel in partial responder patients (PR). 

In cancer, it is well known that lipid and cholesterol homeostasis is often dysregulated. Cancer cells increase lipid demand to facilitate proliferation and evasion from apoptosis. APOL1, a secreted high-density lipoprotein binds to apolipoprotein A-I (ApoA-I), (network 4, Figure 5) which in turn, activates lecithin cholesterol acyl transferase (LCAT) (network 2, Figure 5), leading to the maturation of HDL particles. APOL1 and LCAT were both found to be differentially expressed in our analysis between PR and NR patients, during the treatment (Figure 2b). ApoA-I, a major component of high-density lipoproteins (HDL), is a protein with multifunctional properties, involved in cholesterol trafficking, inflammation, and immune response regulation [66]. Alterations of ApoA-I occur during the development and progression of diverse types of cancer, and a recent discovery showed that ApoA-I was involved in the anti-inflammatory and immune-modulatory mechanisms [66]. Altogether, these results suggest that the complicated lipid and cholesterol homeostasis in cancer cells, tightly regulated through APOL1, LCAT, and ApoA-I, deserve a deeper mechanistic investigation. Investigating the role of these processes might contribute to the improvement of cancer prevention and treatment. 

The metadata analysis approach explained how many proteins of the selected panel were well connected with intracellular/membrane proteins of cancer cells, which connection with 5-FU and leucovorin targets (TYMS and DHFR) and how they were integrated with proteins known to have relevant roles in cancer development such as AKT1 and FOXO3.

## 5. Conclusions

Based on the presented proteomic study, we propose a novel serum protein signature, as a potential predictive response to FOLFOX-4 treatment in patients with heavily pretreated ovarian serous carcinoma. Lipoproteins associated with the metabolic process, structural component modulation in relation to cellular apoptosis and autophagy, and cellular oxidative stress response were identified to be relevant proteins after the multilayer statistical analysis. Our findings were consistent with metadata analysis exploration specifically connected with molecular pathways modulated in ovarian cancer. The results were also supported by the successful network enrichment analysis of the proteins of the final panel with TS and DHFR, a well-known target of FOLFOX-4 components. We noticed that among the 12 final proteins, some were well integrated in the network (such as GSN, LCAT, APOL1, and SHBG), while LYVE1, ROR1, TEC, GFI1, and ZNF573 displayed weak or no interaction. This suggests a certain independent trend among the different proteins of the panel. 

Further analysis showed a metabolic connection between the serum proteins and those belonging to membrane or intracellular networks, and how the proteins of the panel belong to molecular pathways associated with cellular metabolism. The two-time collection analysis of our study supports the concept of the relevance of monitoring circulating proteins during patients’ treatments. The monitoring could also be extended to a higher number of samples collected during therapy. We can conclude that the strategy adopted was successful and the protein panel identified represents an interesting starting point to translate the protein profiles into an exploitable tool for a discovery proteomic work in the near future.

## Figures and Tables

**Table 1:** Statistically significant DEPs between PR versus NR group at time T0 and T1. Up- or downregulation protein abundance was established by mean of a paired *t*-test. Statistically significant *p*-values are reported together with the relative FC (proteins reported in Figure 2a showing *p* < 0.05).

Protein	PR Group log2(T1/T0) Mean FC	NR Group log2(T1/T0) Mean FC	*t* Test *p*-Value	PR/NR Regulation
APOL1	−1.5447	0.5949	0.0156	Downregulation
CO8A	−0.0363	−2.1393	0.0328	Upregulation
FA12	−0.2436	0.9692	0.028	Downregulation
FCGBP	−0.79	1.3221	0.0023	Downregulation
GELS	−0.5374	0.8473	0.0212	Downregulation
HABP2	−0.6816	1.2037	0.0244	Downregulation
IGHG1	−1.2833	0.8845	0.0105	Downregulation
IGM	0.6886	−0.504	0.0345	Upregulation
LCAT	0.8256	−0.1726	0.0345	Upregulation
LMNA	−0.509	0.6646	0.0465	Downregulation
LYVE1	−1.8428	1.1198	0.0051	Downregulation
PGRP2	0.5958	−0.902	0.0079	Upregulation
ROR1	−0.5573	1.0462	0.0361	Downregulation
ZN573	−0.684	1.0665	0.0389	Downregulation

**Table 2:** Proteins selected from those differentially expressed in Table S4 and ANOVA statistic for time and response.

					Interaction	
	Response to Treatment		Timepoint		Response × Timepoint	
Proteins	F Value	*p*-Value	F Value	*p*-Value	F Value	*p*-Value
**APOL1**	0.06	0.8230	1.2	0.3241	12.92	0.0156
**GELS**	4.47	0.0881	1.5	0.2746	10.96	0.0212
**GFI1**	4.06	0.1001	0.27	0.6267	8.6	0.0325
**LCAT**	0.17	0.6939	2.22	0.1967	8.3	0.0345
**LMNA**	2.57	0.1699	0.54	0.4969	6.92	0.0465
**LYVE1**	0.07	0.8054	0.24	0.6471	22.65	0.0051
**ROR1**	0.3	0.6056	2.15	0.2024	10.51	0.0229
**SHBG**	0.01	0.9347	0.16	0.7064	6.74	0.0485
**TEC**	0.1	0.7751	9.81	0.0520	25.57	0.0149
**ZNF573**	0.05	0.8327	1.12	0.3380	8.41	0.0338

## Data Availability

Raw data from the proteomic analysis presented in this study are available at the Fairdomhub publicly accessible repository (doi.org/10.15490/fairdomhub.1.datafile.4074.1). Data file related to the semi-targeted analysis are publicly accessible at the Fairdomhub repository (https://fairdomhub.org/data_files/4088?version=1 (accessed on 18 November 2022).

## Supplementary Materials

The following are available online at https://www.mdpi.com/article/10.3390/cancers15020412/s1, S1. Patients’ characteristics. Table S1. Patients’ characteristics with recurrent ovarian carcinoma included in the translational study. S2. Semitargeted MS analysis for the validation of the protein panel identified with label free MS approach. Table S2. MS parameters for Orbitrap Q-Exactive HRMS validation in the semi-targeted approach. Table S3. Statistical parameters for peptide and protein identification in the semi-targeted approach (Mascot Matrix and Progenesis QI). Table S4. Output parameters of ANOVA applied to the protein identified through label free MS analysis. S5. Statistical approach to protein selection considering timepoints (T0 vs. T1) and differential expression levels. Table S5. Mixed-Effect ANOVA results. Table S6. Biological properties and main interaction description of the protein panel selected from those differentially expressed in Table 2 of the manuscript. S7. Results of the RI Cluster analysis of the 12 DEP’s. Table S7. Results of the RI Cluster analysis of the 12 DEPs. Table S8. Relevant proteins from the local network analysis. Figure S1. Global network visualization based on STRING pathway enrichment analysis of the 14 selected proteins showing the highest scored biological process.

## References

[1] Momenimovahed Z., Tiznobaik A., Taheri S., Salehiniya H. (2019). Ovarian Cancer in the World: Epidemiology and Risk Factors. Int. J. Womens. Health.

[2] Bowtell D.D., Böhm S., Ahmed A.A., Aspuria P.-J., Bast Jr R.C., Beral V., Berek J.S., Birrer M.J., Blagden S., Bookman M.A. (2015). Rethinking Ovarian Cancer II: Reducing Mortality from High-Grade Serous Ovarian Cancer. Nat. Rev. Cancer.

[3] Lorusso D., Fontanella C., Maltese G., Lepori S., Tripodi E., Bogani G., Raspagliesi F. (2017). The Safety of Antiangiogenic Agents and PARP Inhibitors in Platinum-Sensitive Recurrent Ovarian Cancer. Expert Opin. Drug Saf..

[4] Pignata S., Cecere S.C., Du Bois A., Harter P., Heitz F. (2017). Treatment of Recurrent Ovarian Cancer. Ann. Oncol..

[5] Raymond E., Buquet-Fagot C., Djelloul S., Mester J., Cvitkovic E., Allain P., Louvet C., Gespach C. (1997). Antitumor Activity of Oxaliplatin in Combination with 5-Fluorouracil and the Thymidylate Synthase Inhibitor AG337 in Human Colon, Breast and Ovarian Cancers. Anticancer. Drugs.

[6] Pozzi C., Santucci M., Marverti G., D’Arca D., Tagliazucchi L., Ferrari S., Gozzi G., Losi L., Tassone G., Mangani S. (2021). Structural Bases for the Synergistic Inhibition of Human Thymidylate Synthase and Ovarian Cancer Cell Growth by Drug Combinations. Cancers (Basel).

[7] Wilson P.M., Danenberg P.V., Johnston P.G., Lenz H.-J., Ladner R.D. (2014). Standing the Test of Time: Targeting Thymidylate Biosynthesis in Cancer Therapy. Nat. Rev. Clin. Oncol..

[8] Goldberg R.M., Sargent D.J., Morton R.F., Fuchs C.S., Ramanathan R.K., Williamson S.K., Findlay B.P., Pitot H.C., Alberts S.R. (2004). A Randomized Controlled Trial of Fluorouracil Plus Leucovorin, Irinotecan, and Oxaliplatin Combinations in Patients With Previously Untreated Metastatic Colorectal Cancer. J. Clin. Oncol..

[9] Marverti G., Marraccini C., Martello A., D’Arca D., Pacifico S., Guerrini R., Spyrakis F., Gozzi G., Lauriola A., Santucci M. (2021). Folic Acid–Peptide Conjugates Combine Selective Cancer Cell Internalization with Thymidylate Synthase Dimer Interface Targeting. J. Med. Chem..

[10] Niedzwiecki D., Hasson R.M., Lenz H.-J., Ye C., Redston M., Ogino S., Fuchs C.S., Compton C.C., Mayer R.J., Goldberg R.M. (2017). A Study of Thymidylate Synthase Expression as a Biomarker for Resectable Colon Cancer: Alliance (Cancer and Leukemia Group B) 9581 and 89803. Oncologist.

[11] Pectasides D., Pectasides M., Farmakis D., Bountouroglou N., Nikolaou M., Koumpou M., Mylonakis N., Kosmas C. (2003). Oxaliplatin plus High-Dose Leucovorin and 5-Fluorouracil in Pretreated Advanced Breast Cancer: A Phase II Study. Ann. Oncol..

[12] Kim Y.S., Hong J., Sym S.J., Park S.H., Park J., Cho E.K., Lee J.H., Shin D.B. (2010). Oxaliplatin, 5-Fluorouracil and Leucovorin (FOLFOX-4) Combination Chemotherapy as a Salvage Treatment in Advanced Gastric Cancer. Cancer Res. Treat..

[13] de Gramont A., Figer A., Seymour M., Homerin M., Hmissi A., Cassidy J., Boni C., Cortes-Funes H., Cervantes A., Freyer G. (2000). Leucovorin and Fluorouracil With or Without Oxaliplatin as First-Line Treatment in Advanced Colorectal Cancer. J. Clin. Oncol..

[14] Conteduca V., Gurioli G., Rossi L., Scarpi E., Lolli C., Schepisi G., Farolfi A., De Lisi D., Gallà V., Burgio S.L. (2018). Oxaliplatin plus Leucovorin and 5-Fluorouracil (FOLFOX-4) as a Salvage Chemotherapy in Heavily-Pretreated Platinum-Resistant Ovarian Cancer. BMC Cancer.

[15] Wegdam W., Argmann C.A., Kramer G., Vissers J.P., Buist M.R., Kenter G.G., Aerts J.M.F.G., Meijer D., Moerland P.D. (2014). Label-Free LC-MSe in Tissue and Serum Reveals Protein Networks Underlying Differences between Benign and Malignant Serous Ovarian Tumors. PLoS One.

[16] Deng J., Wang L., Ni J., Beretov J., Wasinger V., Wu D., Duan W., Graham P., Li Y. (2016). Proteomics Discovery of Chemoresistant Biomarkers for Ovarian Cancer Therapy. Expert Rev. Proteom..

[17] Agarwal R., Kaye S.B. (2003). Ovarian Cancer: Strategies for Overcoming Resistance to Chemotherapy. Nat. Rev. Cancer.

[18] Babačić H., Lehtiö J., Pico de Coaña Y., Pernemalm M., Eriksson H. (2020). In-Depth Plasma Proteomics Reveals Increase in Circulating PD-1 during Anti-PD-1 Immunotherapy in Patients with Metastatic Cutaneous Melanoma. J. Immunother. Cancer.

[19] Kohli M., Oberg A.L., Mahoney D.W., Riska S.M., Sherwood R., Zhang Y., Zenka R.M., Sahasrabudhe D., Qin R., Zhang S. (2019). Serum Proteomics on the Basis of Discovery of Predictive Biomarkers of Response to Androgen Deprivation Therapy in Advanced Prostate Cancer. Clin. Genitourin. Cancer.

[20] Reymond M.A., Schlegel W. (2007). Proteomics in Cancer. Adv Clin Chem..

[21] Skates S.J., Gillette M.A., LaBaer J., Carr S.A., Anderson L., Liebler D.C., Ransohoff D., Rifai N., Kondratovich M., Težak Ž. (2013). Statistical Design for Biospecimen Cohort Size in Proteomics-Based Biomarker Discovery and Verification Studies. J. Proteome Res..

[22] Tanase C., Albulescu R., Neagu M. (2016). Proteomic Approaches for Biomarker Panels in Cancer. J. Immunoass. Immunochem..

[23] Haymond A., Davis J.B., Espina V. (2019). Proteomics for Cancer Drug Design. Expert Rev. Proteom..

[24] Chinello C., Stella M., Piga I., Smith A.J., Bovo G., Varallo M., Ivanova M., Denti V., Grasso M., Grasso A. (2019). Proteomics of Liquid Biopsies: Depicting RCC Infiltration into the Renal Vein by MS Analysis of Urine and Plasma. J. Proteom..

[25] Therasse P., Arbuck S.G., Eisenhauer E.A., Wanders J., Kaplan R.S., Rubinstein L., Verweij J., Van Glabbeke M., van Oosterom A.T., Christian M.C. (2000). New Guidelines to Evaluate the Response to Treatment in Solid Tumors. JNCI J. Natl. Cancer Inst..

[26] Rustin G.J., Nelstrop A.E., McClean P., Brady M.F., McGuire W.P., Hoskins W.J., Mitchell H., Lambert H.E. (1996). Defining Response of Ovarian Carcinoma to Initial Chemotherapy According to Serum CA 125. J. Clin. Oncol..

[27] Bairoch A. (2000). The SWISS-PROT Protein Sequence Database and Its Supplement TrEMBL in 2000. Nucleic Acids Res..

[28] Liu X., Chinello C., Musante L., Cazzaniga M., Tataruch D., Calzaferri G., James Smith A., De Sio G., Magni F., Zou H. (2015). Intraluminal Proteome and Peptidome of Human Urinary Extracellular Vesicles. PROTEOMICS—Clin. Appl..

[29] Raimondo F., Chinello C., Stella M., Santorelli L., Magni F., Pitto M. (2018). Effects of Hematuria on the Proteomic Profile of Urinary Extracellular Vesicles: Technical Challenges. J. Proteome Res..

[30] Desiere F. (2006). The PeptideAtlas Project. Nucleic Acids Res..

[31] Koenig T., Menze B.H., Kirchner M., Monigatti F., Parker K.C., Patterson T., Steen J.J., Hamprecht F.A., Steen H. (2008). Robust Prediction of the MASCOT Score for an Improved Quality Assessment in Mass Spectrometric Proteomics. J. Proteome Res..

[32] Waters Corporation Progenesis QI for Proteomics. https://www.nonlinear.com/progenesis/qi-for-proteomics/.

[33] Szklarczyk D., Gable A.L., Lyon D., Junge A., Wyder S., Huerta-Cepas J., Simonovic M., Doncheva N.T., Morris J.H., Bork P. (2019). STRING V11: Protein-Protein Association Networks with Increased Coverage, Supporting Functional Discovery in Genome-Wide Experimental Datasets. Nucleic Acids Res..

[34] Bateman A., Martin M.-J., Orchard S., Magrane M., Agivetova R., Ahmad S., Alpi E., Bowler-Barnett E.H., Britto R., Bursteinas B. (2021). UniProt: The Universal Protein Knowledgebase in 2021. Nucleic Acids Res..

[35] Stelzer G., Rosen N., Plaschkes I., Zimmerman S., Twik M., Fishilevich S., Stein T.I., Nudel R., Lieder I., Mazor Y. (2016). The GeneCards Suite: From Gene Data Mining to Disease Genome Sequence Analyses. Curr. Protoc. Bioinforma..

[36] Orre L.M., Vesterlund M., Pan Y., Arslan T., Zhu Y., Fernandez Woodbridge A., Frings O., Fredlund E., Lehtiö J. (2019). SubCellBarCode: Proteome-Wide Mapping of Protein Localization and Relocalization. Mol. Cell.

[37] Binder J.X., Pletscher-Frankild S., Tsafou K., Stolte C., O’Donoghue S.I., Schneider R., Jensen L.J. (2014). COMPARTMENTS: Unification and Visualization of Protein Subcellular Localization Evidence. Database (Oxford).

[38] Thul P.J., Åkesson L., Wiking M., Mahdessian D., Geladaki A., Ait Blal H., Alm T., Asplund A., Björk L., Breckels L.M. (2017). A Subcellular Map of the Human Proteome. Science.

[39] Jassal B., Matthews L., Viteri G., Gong C., Lorente P., Fabregat A., Sidiropoulos K., Cook J., Gillespie M., Haw R. (2020). The Reactome Pathway Knowledgebase. Nucleic Acids Res..

[40] Mi H., Ebert D., Muruganujan A., Mills C., Albou L.-P., Mushayamaha T., Thomas P.D. (2021). PANTHER Version 16: A Revised Family Classification, Tree-Based Classification Tool, Enhancer Regions and Extensive API. Nucleic Acids Res..

[41] Mira E., Carmona-Rodríguez L., Pérez-Villamil B., Casas J., Fernández-Aceñero M.J., Martínez-Rey D., Martín-González P., Heras-Murillo I., Paz-Cabezas M., Tardáguila M. (2018). SOD3 Improves the Tumor Response to Chemotherapy by Stabilizing Endothelial HIF-2α. Nat. Commun..

[42] Sundar S.S., Ganesan T.S. (2007). Role of Lymphangiogenesis in Cancer. J. Clin. Oncol..

[43] Nunomiya K., Shibata Y., Abe S., Inoue S., Igarashi A., Yamauchi K., Kimura T., Aida Y., Nemoto T., Sato M. (2014). Relationship between Serum Level of Lymphatic Vessel Endothelial Hyaluronan Receptor-1 and Prognosis in Patients with Lung Cancer. J. Cancer.

[44] Ose J., Poole E.M., Schock H., Lehtinen M., Arslan A.A., Zeleniuch-Jacquotte A., Visvanathan K., Helzlsouer K., Buring J.E., Lee I.-M. (2017). Androgens Are Differentially Associated with Ovarian Cancer Subtypes in the Ovarian Cancer Cohort Consortium. Cancer Res..

[45] Russell M.R., Graham C., D’Amato A., Gentry-Maharaj A., Ryan A., Kalsi J.K., Ainley C., Whetton A.D., Menon U., Jacobs I. (2017). A Combined Biomarker Panel Shows Improved Sensitivity for the Early Detection of Ovarian Cancer Allowing the Identification of the Most Aggressive Type II Tumours. Br. J. Cancer.

[46] Bertacchini J., Beretti F., Cenni V., Guida M., Gibellini F., Mediani L., Marin O., Maraldi N.M., de Pol A., Lattanzi G. (2013). The protein kinase Akt/PKB regulates both prelamin A degradation and Lmna gene expression. FASEB J..

[47] Hudson M.E., Pozdnyakova I., Haines K., Mor G., Snyder M. (2007). Identification of Differentially Expressed Proteins in Ovarian Cancer Using High-Density Protein Microarrays. Proc. Natl. Acad. Sci. USA.

[48] Yin Z., Gao M., Chu S., Su Y., Ye C., Wang Y., Pan Z., Wang Z., Zhang H., Tong H. (2017). Antitumor Activity of a Newly Developed Monoclonal Antibody against ROR1 in Ovarian Cancer Cells. Oncotarget.

[49] Kipps T. (2020). UC-961 (Cirmtuzumab) in Relapsed or Refractory Chronic Lymphocytic Leukemia.

[50] National Cancer Institute (NCI) (2022). Genetically Modified T-Cell Therapy in Treating Patients With Advanced ROR1+ Malignancies.

[51] Minkenberg R. (2010). Result Disclosure on ClinicalTrials. Gov—First Experiences and Challenges. Pharm. Program..

[52] Abedini M.R., Wang P.-W., Huang Y.-F., Cao M., Chou C.-Y., Shieh D.-B., Tsang B.K. (2014). Cell Fate Regulation by Gelsolin in Human Gynecologic Cancers. Proc. Natl. Acad. Sci. USA.

[53] Asare-Werehene M., Communal L., Carmona E., Le T., Provencher D., Mes-Masson A.-M., Tsang B.K. (2019). Pre-Operative Circulating Plasma Gelsolin Predicts Residual Disease and Detects Early Stage Ovarian Cancer. Sci. Rep..

[54] Griess B., Tom E., Domann F., Teoh-Fitzgerald M. (2017). Extracellular Superoxide Dismutase and Its Role in Cancer. Free Radic. Biol. Med..

[55] Yuan L., Qin X., Li L., Zhou J., Zhou M., Li X., Xu Y., Wang X., Xing H. (2018). The Transcriptome Profiles and Methylation Status Revealed the Potential Cancer-related LncRNAs in Patients with Cervical Cancer. J. Cell Physiol..

[56] Vadnais C., Chen R., Fraszczak J., Hamard P.-J., Manfredi J.J., Möröy T. (2019). A Novel Regulatory Circuit between P53 and GFI1 Controls Induction of Apoptosis in T Cells. Sci. Rep..

[57] Lee C., Rudneva V.A., Erkek S., Zapatka M., Chau L.Q., Tacheva-Grigorova S.K., Garancher A., Rusert J.M., Aksoy O., Lea R. (2019). Lsd1 as a Therapeutic Target in Gfi1-Activated Medulloblastoma. Nat. Commun..

[58] Singh S.S., Vats S., Chia A.Y.-Q., Tan T.Z., Deng S., Ong M.S., Arfuso F., Yap C.T., Goh B.C., Sethi G. (2017). Dual Role of Autophagy in Hallmarks of Cancer. Oncogene.

[59] Segala G., Bennesch M.A., Ghahhari N.M., Pandey D.P., Echeverria P.C., Karch F., Maeda R.K., Picard D. (2019). Vps11 and Vps18 of Vps-C Membrane Traffic Complexes Are E3 Ubiquitin Ligases and Fine-Tune Signalling. Nat. Commun..

[60] Amaravadi R.K., Kimmelman A.C., Debnath J. (2019). Targeting Autophagy in Cancer: Recent Advances and Future Directions. Cancer Discov..

[61] Wan G., Zhaorigetu S., Liu Z., Kaini R., Jiang Z., Hu C.A. (2008). Apolipoprotein L1, a Novel Bcl-2 Homology Domain 3-Only Lipid-Binding Protein, Induces Autophagic Cell Death. J. Biol. Chem..

[62] Taddia L., D’Arca D., Ferrari S., Marraccini C., Severi L., Ponterini G., Assaraf Y.G., Marverti G., Costi M.P. (2015). Inside the Biochemical Pathways of Thymidylate Synthase Perturbed by Anticancer Drugs: Novel Strategies to Overcome Cancer Chemoresistance. Drug Resist. Updat..

[63] Genovese F., Gualandi A., Taddia L., Marverti G., Pirondi S., Marraccini C., Perco P., Pelà M., Guerrini R., Amoroso M.R. (2014). Mass Spectrometric/Bioinformatic Identification of a Protein Subset That Characterizes the Cellular Activity of Anticancer Peptides. J. Proteome Res..

[64] Wang Y., Wang Q., Huang H., Huang W., Chen Y., McGarvey P.B., Wu C.H., Arighi C.N. (2021). A Crowdsourcing Open Platform for Literature Curation in UniProt. PLOS Biol..

[65] Reimand J., Isserlin R., Voisin V., Kucera M., Tannus-Lopes C., Rostamianfar A., Wadi L., Meyer M., Wong J., Xu C. (2019). Pathway Enrichment Analysis and Visualization of Omics Data Using g:Profiler, GSEA, Cytoscape and EnrichmentMap. Nat. Protoc..

[66] Georgila K., Vyrla D., Drakos E. (2019). Apolipoprotein A-I (ApoA-I), Immunity, Inflammation and Cancer. Cancers (Basel).

[67] Gonzalez-Angulo A.M., Hennessy B.T., Meric-Bernstam F., Sahin A., Liu W., Ju Z., Carey M.S., Myhre S., Speers C., Deng L. (2011). Functional Proteomics Can Define Prognosis and Predict Pathologic Complete Response in Patients with Breast Cancer. Clin. Proteom..

[68] Gonzalez-Angulo A.M., Liu S., Chen H., Chavez-MacGregor M., Sahin A., Hortobagyi G.N., Mills G.B., Do K.-A., Meric-Bernstam F. (2013). Functional Proteomics Characterization of Residual Breast Cancer after Neoadjuvant Systemic Chemotherapy. Ann. Oncol..

[69] Stearns V., Singh B., Tsangaris T., Crawford J.G., Novielli A., Ellis M.J., Isaacs C., Pennanen M., Tibery C., Farhad A. (2003). A Prospective Randomized Pilot Study to Evaluate Predictors of Response in Serial Core Biopsies to Single Agent Neoadjuvant Doxorubicin or Paclitaxel for Patients with Locally Advanced Breast Cancer. Clin. Cancer Res..

[70] Asem M.S., Buechler S., Wates R.B., Miller D.L., Stack M.S. (2016). Wnt5a Signaling in Cancer. Cancers (Basel).

[71] Yin Z., Mao Y., Zhang N., Su Y., Zhu J., Tong H., Zhang H. (2019). A Fully Chimeric IgG Antibody for ROR1 Suppresses Ovarian Cancer Growth in Vitro and in Vivo. Biomed. Pharmacother..

[72] Timms J.F., Arslan-Low E., Kabir M., Worthington J., Camuzeaux S., Sinclair J., Szaub J., Afrough B., Podust V.N., Fourkala E.-O. (2014). Discovery of Serum Biomarkers of Ovarian Cancer Using Complementary Proteomic Profiling Strategies. Proteomics. Clin. Appl..

[73] Lopez-Bergami P., Barbero G. (2020). The Emerging Role of Wnt5a in the Promotion of a Pro-Inflammatory and Immunosuppressive Tumor Microenvironment. Cancer Metastasis Rev..

[74] Peng C., Zhang X., Yu H., Wu D., Zheng J. (2011). Wnt5a as a Predictor in Poor Clinical Outcome of Patients and a Mediator in Chemoresistance of Ovarian Cancer. Int. J. Gynecol. Cancer.

[75] Varma R., Hector S., Clark K., Greco W., Hawthorn L., Pendyala L. (2005). Gene Expression Profiling of a Clonal Isolate of Oxaliplatin-Resistant Ovarian Carcinoma Cell Line A2780/C10. Oncol. Rep..

