# Supplementary material for: Serum Mass Spectrometry Proteomics and Protein Set Identification in Response to FOLFOX-4 in Drug-Resistant Ovarian Carcinoma
Source: Cancers (Basel). 2023 Jan 8;15(2):412. doi: 10.3390/cancers15020412 (PMC9856519; doi:10.3390/cancers15020412)
Supplement: Supplementary file 1 [file cancers-15-00412-s001.zip › cancers-2116768-supplementary.pdf]

Article

Serum Mass Spectrometry Proteomics and Protein Set Identification in Response to FOLFOX-4 in Drug-Resistant Ovarian Carcinoma

Domenico D'Arca, Leda Severi, Stefania Ferrari, Luca Dozza, Gaetano Marverti, Fulvio Magni, Clizia Chinello, Lisa Pagani, Lorenzo Tagliazucchi, Marco Villani, Gianluca d'Addese, Isabella Piga, Vincenza Conteduca, Lorena Rossi, Giorgia Gurioli, Ugo De Giorgi, Lorena Losi and Maria Paola Costi

S1. Patients' characteristics with recurrent ovarian carcinoma included in the translational study

FOLFOX-4 consists of 85 mg/m^2 of oxaliplatin as a 2-hour infusion on day 1, 200 mg/m^2 of LV (leucovorin) as a 2-hour infusion on day 1, and a bolus 400 mg/m^2 of 5-FU on day 1, followed by a 22-hour infusion of 600 mg/m^2 of 5-FU for two consecutive days every three weeks. Chemotherapy was terminated when progressive disease developed. The most frequent chemotherapy-induced adverse event was neutropenia, which was controlled with as the administration of granulocyte colony stimulating factor, or with chemotherapy delay and/or dose reduction. Tumor response was assessed every three cycles by repeating baseline assessments using imaging studies (computed axial tomography CAT) according to the Response Evaluation Criteria in Solid Tumors (RECIST) for patients with measurable disease.

Partial response (PR) or not response (NR) were defined as a reduction of more than 30% of the sum of the perpendicular diameters of lesions, and the absence of any tumor response, respectively. In addition, tumor response for patients with non-measurable disease was also assessed according to the validated cancer antigen 125 (CA-125) criteria proposed by Rustin. All the patients shared the following characteristics from baseline serum collection (T0) to T1 collection: i. no progression of the disease after the first cycle of FOLFOX-4 administration (T1); ii. no adverse effect reported after T1; iii. Distinction between PR and NR was assessed as previously mentioned, with a follow-up evaluation 6-months after FOLFOX-4 cycle.

Table S1. Patients' characteristics with recurrent ovarian carcinoma, included in the translational study. Light green highlight shows the patients whose serum sample was not selected for further analysis.

Patient	Outcome	Previous Pharmacological treatments	Cycles of FOLFOX-4
F1	NR	carbo+taxol; carbo+caelyx; topotecan; taxol; gemcitabine; etoposide; cyclophosphamide	6
F2	NR	carbo+taxol; HIPEC; carbo+taxol; carboplatin; caelyx	2
F3	PR	carbo+taxol; caelyx; topotecan	7
F4	NR	carbo+taxol; tamoxifen; caelyx; cisplatin+vinorelbine; topotecan; melphalan	4
F7	PR	carbo+taxol; caelyx; topotecan; anastrozole	17
F8	NR	carbo+taxol; carbo+taxol; caelyx+gemcitabine	4
F9	NR	carbo+taxol; HIPEC; caelyx+gemcitabine	2
F10	PR	carbo+taxol; carbo+gemcitabine; trabectedine+caelyx; carboplatin; taxol	10
F11	NR	carbo+taxol; caelyx; melphalan	1
F12	NR	carbo+taxol; carbo+taxol; cisplatin+gemcitabine; gemcitabine	4

S2. Semitargeted MS analysis for the validation of the protein panel identified with label free MS approach

The two most differing samples for the 12 DEP's for $t_{(0)}$ F10 and F12, were chosen to replicate the experiment run at the University of Milano Bicocca (Monza) on Impact Bruker QqTOF. Samples were treated as reported in main text, chapter 2.3, to eliminate the HAP's. An SDS-page was run on each fraction (LAP's, intermediate and HAP's) to evaluate the quality of the immunodepletion step. LAP's concentrations were assessed with a Bradford micro-assay performed with Coomassie Dye Reagent 1x (BioRad) on a 96 well-plate with a Thermo Scientific™ Multiskan™ GO Microplate Spectrophotometer

A FASP standard protocol (Filter Aided Sample Preparation) was performed as described in Section 2.4, main text. Samples were resuspended in 97% water, 2% ACN + 1% formic acid and sonicated for 15 minutes in a warm bath. A C18 Hypersil GOLD™ (Thermo Fisher™) column, 100 x 2.1mm, 1.9um particle size was employed for peptide separation on an UHPLC UltiMate 3000 system (Thermo Fisher) coupled to a Orbitrap Q-Exactive HRMS (Thermo Fisher). The so defined *.raw files'* obtained from Thermo Fisher system were converted into *.mgf* extension to be analysed with Mascot Studio software. A preliminary quantitative assessment was established to determine the number of proteins matches of the examined samples. p-val <0.05%, p-val <0.01% and a p-value adjusted to FDR 5% (against a reversed-sequence simulated decoy database) on protein identification were considered- separately to upgrade instrumental performances basing on the number of proteins matched and peptides found.

For the quantification, only the two most abundant non-conflicting (or 'unique') peptides were considered to avoid sequence-overlapping peptides and false positive quantitation. The sum of the peptide areas with the isotopic pattern was calculated by the software. Area values were normalized and exported to an CSV file. The Fold Change (FC) was calculated by dividing normalized area of F10 mean areas and F12 mean areas. A p-value vas assigned to each protein according to Progenesis Suite and Mascot Scoring System, considering i. Coherence between the replicate; ii. The number of MS queries for each peptide; iii MS/MS threshold mass tolerance. The addition of an inclusion list with the m/z of the most observable peptides (PeptideAtlas) allowed to quantify the proteins through the sole most representative peptides allowed to elude the score matching process and the instrumental sensitivity.

Results were analysed with a protocol for a semi-targeted experiment: the peak list for the peptides of interest was extracted and confronted to the theoretical MS/MS values with Progenesis QI for Proteomics™ software.

Table S2. MS parameters for Orbitrap Q-Exactive HRMS validation in the semi-targeted approach.

Resolution	70.000
ACG target	3,00E+06
Max IT	243 ms
Range	200 to 2000 m/Z
Spectrum	Centroid
**MS/MS**	
Resolution	17.500
ACG target	2,00E+05
Max IT	120ms
Loop Count	8
Top N	8
Isolation window	2.3 m/Z
nce	28
Spectrum	Centroid
Min ACG	1,30E+03
Intensity threshold	1,10E+04
Apex trigger	1 to 24 s
Charge excl.	1; unassigned
Dynamic excl.	60 s

Table S3. Statistical parameters for peptide and protein identification in the semi-targeted approach.

Databases	SwissProt
	cRAP (contaminants)
**MS tolerance**	10 ppm
**MS/MS tolerance**	0.02 Da
**Fixed modifications**	Carbamidomethylation (C)
**Variable modifications**	Oxidation (M)
	Deamination (N, Q)
**Digestion**	Trypsin
**Max missed cleavages**	1

Table S4. Output parameters of ANOVA applied to the protein identified through label free MS analysis. The F statistics is related to the 3 covariates (R=response, T=Time, Int=Interaction). Relevant proteins are yellow (timepoint and response) and green (only response) highlighted.

Protein list	IInt_F_Value	Int_pvalue	R_F_Value	R_pvalue	T_F_Value	T_pvalue	RT_F_Value	RT_pvalue
AFAM_HUMAN	80947.2	1.0179856957393e-11	2.5	0.174688978	6.89	0.04681126	2.14	0.203220537
APOD_HUMAN	87355.04	8.41460234823899e-12	1.05	0.353296028	7.64	0.039669187	0.07	0.797139392
APOL1_HUMAN	19648.67	3.50537265880746e-10	0.06	0.82299487	1.2	0.324108834	12.92	0.015632474
CBG_HUMAN	44756.73	4.4776848895367e-11	0.82	0.407844372	8.38	0.03398298	0.63	0.464588141
CFAI_HUMAN	127068.54	3.29736238313671e-12	10.88	0.021518392	0.59	0.477567534	7.65	0.039591092
CO5_HUMAN	100456.05	5.93358695510915e-12	0.29	0.611808341	9.08	0.029652299	0.98	0.368200084
CO7_HUMAN	236321.51	6.98996416303999e-13	2.68	0.162829818	18.15	0.008014701	1.08	0.346737181
CO8A_HUMAN	81294.75	1.00713881678871e-11	3.58	0.11723312	13.12	0.015190835	9.27	0.028598037
CO9_HUMAN	308200.18	3.59934304583476e-13	0.64	0.46026843	16.69	0.009487117	3.03	0.142392689
F13B_HUMAN	79433.64	1.06716857573019e-11	0.29	0.612588085	10.88	0.021524854	0.42	0.546517368
FA12_HUMAN	235137.17	7.07878200501e-13	0.69	0.444737841	5.26	0.070338698	9.38	0.028009259
FA9_HUMAN	96669.91	6.53166409847472e-12	1.79	0.238534557	6.33	0.053386326	8.12	0.035813465
FCGBP_HUMAN	180884.76	1.36379796344954e-12	0.07	0.803511414	5.21	0.071287834	32.76	0.002277794
GELS_HUMAN	45135.47	4.38435954208671e-11	4.47	0.088106842	1.5	0.274585582	10.96	0.021218884
GFI1_HUMAN	38922.21	6.34878816185847e-11	4.06	0.100096738	0.27	0.626661529	8.6	0.032542128
HABP2_HUMAN	126387.01	3.34199334872665e-12	0.01	0.907655878	2.16	0.201969136	11.98	0.018009774
HBA_HUMAN	38194.14	6.65564270363461e-11	0.25	0.638536161	9.15	0.029236702	0.14	0.722981916
HEP2_HUMAN	67110.64	1.62651003776659e-11	1.21	0.321559283	7.11	0.044497177	0.16	0.706047516
HGFA_HUMAN	22825.8	2.41009878720888e-10	1.08	0.345995678	13.52	0.01433004	0.13	0.731813263
HPT_HUMAN	39798.42	6.00512972681599e-11	0.43	0.538995699	9.52	0.02731452	0.15	0.716333292

HV601_HU-MAN	27810.16	1.47105216896648e-10	0.01	0.911603577	7.45	0.041297647	0.02	0.881579359
IC1_HU-MAN	147920.95	2.25530705222354e-12	2.64	0.165215209	10.86	0.021593516	0.06	0.8200584
IGHA1_HU MAN	31935.16	1.0410827755436e-10	1.05	0.352088521	16.71	0.009464218	0.02	0.902922017
IGHG1_HU MAN	47854.13	3.78799214217906e-11	0	0.951316302	0.03	0.874928396	15.9	0.010456562
IGJ_HU-MAN	132877.93	2.94875235340442e-12	0.07	0.801513453	9.32	0.028347308	1.6	0.261662743
IGK_HU-MAN	71784.57	1.37454492232791e-11	0.26	0.630689449	8.68	0.032049287	2.95	0.14648397
IGM_HU-MAN	41610.35	5.37265787414754e-11	1.81	0.236228842	0	0.973537217	8.3	0.03453168
ITIH1_HU-MAN	99971.35	6.00575145170978e-12	0.42	0.546663642	8.64	0.032284703	0.15	0.718901985
ITIH4_HU-MAN	167543.85	1.65178981603731e-12	0.27	0.62556993	8.13	0.035733323	0.12	0.741988576
K22E_HU-MAN	92542.49	7.28450633147304e-12	0.04	0.852254108	8.09	0.036070529	0.08	0.791755754
K2C1_HU-MAN	122072.96	3.64508423444931e-12	12.74	0.016059585	1.24	0.315781969	4.97	0.076213788
KR131_HU-MAN	59787.75	2.17117435141745e-11	7.83	0.038052162	0.08	0.790968767	0	0.956241958
LCAT_HU-MAN	72882.63	1.32336364089269e-11	0.17	0.693897244	2.22	0.196672608	8.3	0.034518551
LMNA_HU-MAN	150603.96	2.15616413612452e-12	2.57	0.169894657	0.54	0.496913613	6.92	0.046519446
LUM_HU-MAN	30792.26	1.140377792197e-10	0.02	0.902663765	7.4	0.041790848	1.13	0.337341148
LYVE1_HU-MAN	36319.79	7.54774021061166e-11	0.07	0.805410563	0.24	0.647129975	22.65	0.00506156
PGRP2_HU-MAN	38537.05	6.50859366402301e-11	0.07	0.80172167	2.25	0.193946501	18.27	0.007906172
PZP_HU-MAN	19502.36	3.57147200702457e-10	0	0.948191469	8.88	0.030817572	0.1	0.760896095
ROR1_HU-MAN	152054.97	2.10509387699176e-12	0.3	0.605559738	2.15	0.202422907	10.51	0.022907104
SAA4_HU-MAN	72159.79	1.35675914947342e-11	0.98	0.36682888	33.11	0.002224092	0.39	0.562048575
SAMP_HU-MAN	61512.21	2.02220462597325e-11	1.28	0.309940478	0.01	0.927097186	7.98	0.03692174
SHBG_HU-MAN	27696.19	1.48622891771311e-10	0.01	0.934667246	0.16	0.70644185	6.74	0.048485643
SODE_HU-MAN	84601.58	9.11604125519716e-12	9.26	0.028626263	0.04	0.842464443	1.5	0.274565664
TEC_HU-MAN	118370.07	5.41495284167581e-08	0.1	0.775137594	9.81	0.051969737	25.57	0.014920849
TITIN_HU-MAN	310545.15	3.53161944133262e-13	0	0.960332252	7.07	0.044909485	3.93	0.104168007
TRFE_HU-MAN	76222.36	1.18314247288254e-11	2.99	0.144152128	34.52	0.002027783	5.1	0.073450107
TSP1_HU-MAN	23528.79	2.2341239969137e-10	0.12	0.74008148	8.09	0.036062687	0.26	0.633604919
VPS18_HU-MAN	186976.14	1.25544019624613e-12	14.48	0.012565355	2.58	0.169401263	1.22	0.318932927

VTNC_HUMAN	68677.29	1.53532742075413e-11	0.37	0.571038773	9.07	0.029685547	0.6	0.472912744
ZN573_HUMAN	102526.15	5.63860069746625e-12	0.05	0.832688379	1.12	0.338022242	8.41	0.033765464

S5. Statistical approach to protein selection considering timepoints (T0 vs T1) and differential expression levels.

Response to treatment has a statistically significant contribution on differential expression of 5 proteins (Table S5a), otherwise protein expression of 27 proteins was identified mainly driven independently by timepoint (Table S5b), lastly 20 proteins have demonstrated an iterative effect of treatment response and timepoint (Table S5c) on their differential expression.

Table S5. Mixed Effect ANOVA results.

**a**	**Response to treatment**		**Timepoint**		**Interaction Response x Timepoint**	
**Proteins**	**F Value**	**P-Value**	**F Value**	**P-Value**	**F Value**	**P-Value**
**CFAI_HUMAN**	10.88	0.0215	0.59	0.4776	7.65	0.0396
**K2C1_HUMAN**	12.74	0.0161	1.24	0.3158	4.97	0.0762
**KR131_HUMAN**	7.83	0.0381	0.08	0.7910	0	0.9562
**SOD3_HUMAN**	9.26	0.0286	0.04	0.8425	1.5	0.2746
**VPS18_HUMAN**	14.48	0.0126	2.58	0.1694	1.22	0.3189

**b**	**Response to treatment**		**Timepoint**		**Interaction Response x Timepoint**	
**Proteins**	**F Value**	**P-Value**	**F Value**	**P-Value**	**F Value**	**P-Value**
**AFAM_HUMAN**	2.5	0.1747	6.89	0.0468	2.14	0.2032
**APOD_HUMAN**	1.05	0.3533	7.64	0.0397	0.07	0.7971
**CBG_HUMAN**	0.82	**0.**4078.	8.38	0.0340	0.63	0.4646
**CO5_HUMAN**	0.29	0.6118	9.08	0.0297	0.98	0.3682
**CO7_HUMAN**	2.68	0.1628	18.15	0.0080	1.08	0.3467
**CO8A_HUMAN**	3.58	0.1172	13.12	0.0152	9.27	0.0286
**CO9_HUMAN**	0.64	0.4603	16.69	0.0095	3.03	0.1424
**F13B_HUMAN**	0.29	0.6126	10.88	0.0215	0.42	0.5465
**HBA_HUMAN**	0.25	0.6385	9.15	0.0292	0.14	0.7230
**HEP2_HUMAN**	1.21	0.3216	7.11	0.0445	0.16	0.7060
**HGFA_HUMAN**	1.08	0.3460	13.52	0.0143	0.13	0.7318
**HPT_HUMAN**	0.43	0.5390	9.52	0.0273	0.15	0.7163
**HV601_HUMAN**	0.01	0.9116	7.45	0.0413	0.02	0.8816
**IC1_HUMAN**	2.64	0.1652	10.86	0.0216	0.06	0.8201
**IGHA1_HUMAN**	1.05	0.3521	16.71	0.0095	0.02	0.9029
**IGJ_HUMAN**	0.07	0.8015	9.32	0.0283	1.6	0.2617
**IGK_HUMAN**	0.26	0.6307	8.68	0.0320	2.95	0.1465

**ITIH1_HUMAN**	0.42	0.5467	8.64	0.0323	0.15	0.7189
**ITIH4_HUMAN**	0.27	0.6256	8.13	0.0357	0.12	0.7420
**K22E_HUMAN**	0.04	0.8523	8.09	0.0361	0.08	0.7918
**LUM_HUMAN**	0.02	0.9027	7.4	0.0418	1.13	0.3373
**PZP_HUMAN**	0	0.9482	8.88	0.0308	0.1	0.7609
**SAA4_HUMAN**	0.98	0.3668	33.11	0.0022	0.39	0.5620
**TITIN_HUMAN**	0	0.9603	7.07	0.0449	3.93	0.1042
**TRFE_HUMAN**	2.99	0.1442	34.52	0.0020	5.1	0.0735
**TSP1_HUMAN**	0.12	0.7401	8.09	0.0361	0.26	0.6336
**VTNC_HUMAN**	0.37	0.5710	9.07	0.0297	0.6	0.4729

**c**	**Response to treatment**		**Timepoint**		**Interaction Response x Timepoint**	
**Proteins**	**F Value**	**P-Value**	**F Value**	**P-Value**	**F Value**	**P-Value**
**APOL1_HUMAN**	0.06	0.8230	1.2	0.3241	12.92	0.0156
**CFAI_HUMAN**	10.88	0.0215	0.59	0.4776	7.65	0.0396
**CO8A_HUMAN**	3.58	0.1172	13.12	0.0152	9.27	0.0286
**FA12_HUMAN**	0.69	0.4447	5.26	0.0703	9.38	0.0280
**FA9_HUMAN**	1.79	0.2385	6.33	0.0534	8.12	0.0358
**FCGBP_HUMAN**	0.07	0.8035	5.21	0.0713	32.76	0.0023
**GELS_HUMAN**	4.47	0.0881	1.5	0.2746	10.96	0.0212
**GFI1_HUMAN**	4.06	0.1001	0.27	0.6267	8.6	0.0325
**HABP2_HUMAN**	0.01	0.9077	2.16	0.2020	11.98	0.0180
**IGHG1_HUMAN**	0	0.9513	0.03	0.8749	15.9	0.0105
**IGM_HUMAN**	1.81	0.2362	0	0.9735	8.3	0.0345
**LCAT_HUMAN**	0.17	0.6939	2.22	0.1967	8.3	0.0345
**LMNA_HUMAN**	2.57	0.1699	0.54	0.4969	6.92	0.0465
**LYVE1_HUMAN**	0.07	0.8054	0.24	0.6471	22.65	0.0051
**PGRP2_HUMAN**	0.07	0.8017	2.25	0.1939	18.27	0.0079
**ROR1_HUMAN**	0.3	0.6056	2.15	0.2024	10.51	0.0229
**SAMP_HUMAN**	1.28	0.3099	0.01	0.9271	7.98	0.0369
**SHBG_HUMAN**	0.01	0.9347	0.16	0.7064	6.74	0.0485
**TEC_HUMAN**	0.1	0.7751	9.81	0.0520	25.57	0.0149
**ZN573_HUMAN**	0.05	0.8327	1.12	0.3380	8.41	0.0338

Table S6. Biological properties and main interaction description of the protein panel selected from those differentially expressed in Table 2 of the manuscript.

Protein code (Uniprot)	Protein Function
**APOL1**	This gene encodes a secreted high-density lipoprotein which binds to apolipoprotein A-I. Apolipoprotein A-I is a relatively abundant plasma protein and is the major apoprotein of HDL. It is involved in the formation of most cholesteryl esters in plasma and also promotes efflux of cholesterol from cells. This apolipoprotein L family member may play a role in lipid exchange and transport throughout the body, as well as in reverse cholesterol transport from peripheral cells to the liver. CETP (Cholesteryl Ester Transfer Protein, Plasma) is a Protein Coding gene. Gene Ontology (GO) annotations related to this gene include *lipid binding* and *lipid transporter activity*. ACTA1 The product encoded by this gene belongs to the actin family of proteins, which are highly conserved proteins that play a role in cell motility, structure and integrity. APOL1-(CETP-LCAT-LPA-APOA1),APOA1-GSN-ACTA1
**GSN**	Gelsolin; Calcium-regulated, actin-modulating protein that binds to the plus (or barbed) ends of actin monomers or filaments, preventing monomer exchange (end-blocking or capping). It can promote the assembly of monomers into filaments (nucleation) as well as sever filaments already formed. Plays a role in ciliogenesis; Gelsolin/villins (782 aa). The protein encoded by this gene binds to the "plus" ends of actin monomers and filaments to prevent monomer exchange. The encoded calcium-regulated protein functions in both assembly and disassembly of actin filaments. The localization is extracellular (5/5), GeneCards.
**GFI1**	Zinc finger protein Gfi-1; Transcription repressor essential for hematopoiesis. Functions in a cell-context and development-specific manner. Binds to 5'-TAAATCAC[AT]GCA-3' in the promoter region of a large number of genes. Component of several complexes, including the EHMT2- GFI1-HDAC1, AJUBA-GFI1-HDAC1 and RCOR-GFI-KDM1A-HDAC complexes, that suppress, via histone deacetylase (HDAC) recruitment, a number of genes implicated in multilineage blood cell development. (422 aa). **Growth factor independent transcriptional repressor.** This gene encodes a nuclear zinc finger protein that functions as a transcriptional repressor. This protein plays a role in diverse developmental contexts, including hematopoiesis and oncogenesis. It functions as part of a complex along with other cofactors to control histone modifications that lead to silencing of the target gene promoters. Mutations in this gene cause autosomal dominant severe congenital neutropenia, and also dominant nonimmune chronic idiopathic neutropenia of adults, which are heterogeneous hematopoietic disorders that cause predispositions to leukemias and infections. Multiple alternatively spliced variants, encoding the same protein, have been identified for this gene. [provided by RefSeq, Jul 2008]. Found specifically in bone marrow. (BRCA1-**AKT1-TP53-GFI1**-HDAC1) (-HDAC1**AKT1**-FOXO3-BRCA1) (FOXO3-TP53-GFI1). **General blood factor growth up-regulated in cancer progression** (GeneCards-STRING). Localization in the nucleus (level 5/5) and extracellular (level 2/5). Average expression in ovary.
**LCAT**	Phosphatidylcholine-sterol acyltransferase; Central enzyme in the extracellular metabolism of plasma lipoproteins. Synthesized mainly in the liver and secreted into plasma where it converts cholesterol and phosphatidylcholines (lecithins) to cholesteryl esters and lysophosphatidylcholines on the surface of high and low density lipoproteins (HDLs and LDLs). The cholesterol ester is then transported back to the liver. Has a preference for plasma 16-0-18-2 or 18-O-18-2 phosphatidylcholines. (440 aa). LCAT-LPA(LPA)-APOA1(SHGB) -CETP- IL6 (SHGB).
**LMNA**	Prelamin-A/C; Lamins are components of the nuclear lamina, a fibrous layer on the nucleoplasmic side of the inner nuclear membrane, which is thought to provide a framework for the nuclear envelope and may also interact with chromatin. Lamin A and C are present in equal amounts in the lamina of mammals. Plays an important role in nuclear assembly, chromatin organization, nuclear membrane and telomere dynamics. Required for normal development of peripheral nervous system and skeletal muscle and for muscle satellite cell proliferation. Required for osteoblastogenesis and bone formation. (664 aa). VCL-AKT1-LMNA -CDK1
**LYVE1**	Lymphatic vessel endothelial hyaluronic acid receptor 1; Ligand-specific transporter trafficking between intracellular organelles (TGN) and the plasma membrane. Plays a role in autocrine regulation of cell growth mediated by growth regulators containing cell surface retention sequence binding (CRS). May act as a hyaluronan (HA) transporter, either mediating its uptake for catabolism within lymphatic endothelial cells themselves, or its transport into the lumen of afferent lymphatic vessels for subsequent re-uptake and degradation in lymph nodes (322 aa). This gene encodes a type I integral membrane glycoprotein. The encoded protein acts as a receptor and binds to both soluble and immobilized hyaluronan. This protein may function in lymphatic

	hyaluronan transport and have a role in tumor metastasis. [provided by RefSeq, Jul 2008]. Soluble protein. LYVE1-STAB2-TMSB4X-ACTA1-AKT1 (GeneCards, STRING). TMSB4X is a protein coding gene Thymosin Beta 4 X-Linked), actin sequestering protein which plays a role in regulation of actin polymerization. The protein is also involved in cell proliferation, migration, and differentiation.
**ROR1**	Inactive tyrosine-protein kinase transmembrane receptor ROR1; Has very low kinase activity in vitro and is unlikely to function as a tyrosine kinase in vivo. Receptor for ligand WNT5A which activate downstream NFkB signaling pathway and may result in the inhibition of WNT3A-mediated signaling. In inner ear, crucial for spiral ganglion neurons to innervate auditory hair cells; I-set domain containing (937 aa) (Figure 1a)
**SHBG**	Sex hormone-binding globulin; Functions as an androgen transport protein, but may also be involved in receptor mediated processes. Each dimer binds one molecule of steroid. Specific for 5-alpha-dihydrotestosterone, testosterone, and 17-beta-estradiol. Regulates the plasma metabolic clearance rate of steroid hormones by controlling their plasma concentration (402 aa). LCAT-LPA(LPA)-APOA1(SHGB) -CETP- IL6 (SHGB)-AKT1(APOA1). Almost all proteins are involved in cholesterol metabolism.
**SOD3 (SODE)**	Extracellular superoxide dismutase [Cu-Zn]; Protect the extracellular space from toxic effect of reactive oxygen intermediates by converting superoxide radicals into hydrogen peroxide and oxygen (240 aa). SOD1-SOD3-FOXO3-(AKT1)
**TEC**	Tyrosine-protein kinase Tec; Non-receptor tyrosine kinase that contributes to signaling from many receptors and participates as a signal transducer in multiple downstream pathways, including regulation of the actin cytoskeleton. Plays a redundant role to ITK in regulation of the adaptive immune response. Regulates the development, function and differentiation of conventional T-cells and nonconventional NKT-cells. Required for TCR-dependent IL2 gene induction. (631 aa). ITK-LAT-(WAS)-MAP4K1. WASL linked to WAS linked to WIPF2 (WAS/WASL-interacting protein family member 2; Plays an active role in the formation of cell surface protrusions downstream of activated PDGFB receptors. Plays an important role in actin-microspike formation through cooperation with WASL. May cooperate with WASP and WASL to induce mobilization and reorganization of the actin filament system). WASL is connected with WNT5A and ROR1. Localization: plasma membrane and extracellular (GeneCards). Based on Proteomic database evidence, it has been found in Peripheral blood mononuclear cells, and in platelets. (ROR1-WNT5A-WAS-TEC)
**VPS18**	Vacuolar protein sorting-associated protein 18 homolog; Plays a role in vesicle-mediated protein trafficking to lysosomal compartments including the endocytic membrane transport and autophagic pathways. Believed to act as a core component of the putative HOPS and CORVET endosomal tethering complexes which are proposed to be involved in the Rab5-to-Rab7 endosome conversion probably implicating MON1A/B, and via binding SNAREs and SNARE complexes to mediate tethering and docking events during SNARE-mediated membrane fusion. This protein has been identified by us for the first time.
**ZNF573**	Zinc finger protein 573; May be involved in transcriptional regulation; Zinc fingers C2H2-type (665 aa). Diseases associated with ZNF573 include Endocervical Carcinoma and Endocervical Adenocarcinoma. Among its related pathways are Gene Expression. Gene Ontology (GO) annotations related to this gene include *nucleic acid binding* and *DNA-binding transcription factor activity*. An important paralog of this gene is ZNF607. Despite its localization in the nucleus and cytosol (5 and 4/5, only 1/5 in the extracellular space, its estimated expression is positive in plasma and platelet and also in ovary cells. This gene is overexpressed in Ovary (21.2), Plasma (19.1), Spleen (17.9), and Testis (9.8). (GeneCards). The local network: ZNF573-TRIM28(CDC5L-ATM) is supported by co-expression experimental evidence and database. There is not much evidence to correlate specific serum protein identification and cellular network.

S7. Results of the RI Cluster analysis of the 12 DEP's

RI analysis is here applied to evaluate if proteins relate to each other according to their variables (FC and p-value) to generate clusters. By considering each triplicate, the clusters LCAT+LMNA and APOL1+LYVE1 always appear (with or without other terms). If the same RI analysis is applied after the removal of the outlier data (Q-test 95%), the number of observations decreases but the cluster APOL1 + LYVE1 is still present. On the other hand, no observation is registered If a geometrical mean of triplicate is employed instead of the outlier data.

Table S7. Details of the RI Cluster analysis of the 12 DEP's.

Considering all the values for MS triplicates (normalized area values)			
**Basal only (zImax=45.65)**		**Co-variate (T0 vs T1) (zImax=45.65)**	
**APOL1 / GFI1 / LYVE1**	zI= 4.20	**GFI1 / LCAT / LMNA**	zI= 3.67
**LCAT / LMNA**	zI= 3.25		
Initial values (without outliers, i.e. after Q test 95% confidence interval, the outliers values are eliminated)			
**Basal only (zImax= 45.65)**			
**APOL1 / GFI1 / LYVE1**	zI= 3.18		

Table S8. Relevant proteins from the local network analysis.

Protein Code	Protein Significance	Protein Connection with Ovarian Cancer	Reference in Ovarian Cancer
**AKT1**	AKT1, Alpha Serine/Threonine Kinase 1, also referred to as protein kinase B, is a known oncogene. AKT activation relies on the PI3K pathway and is recognized as a critical node in the pathway. The E17 hotspot is the most characterized of AKT1 mutations and has been shown to result in activation of the protein. Mutations in AKT1 have also been shown to confer resistance to allosteric kinase inhibitors in vitro.	Akt is a serine-threonine kinase that is overexpressed in numerous cancers, including ovarian. AKT1 is involved in a high number of signalling (AKT1 signalling pathway). It is a anticancer target.	Linnerth-Petrik N. M., Santry L. A., Moorehead R., Jücker M., Wootton S. K., Petrik J. Akt isoform specific effects in ovarian cancer progression. Oncotarget. 2016; 7: 74820-74833.
**ACTA1**	ACTA1, Actin Alpha 1. The product encoded by this gene belongs to the actin family of proteins, which are highly conserved proteins that play a role in cell motility, structure and integrity. Alpha, beta and gamma actin isoforms have been identified, with alpha actins being a major constituent of the contractile apparatus, while beta and gamma actins are involved in the regulation of cell motility. This actin is an alpha actin that is found in skeletal muscle.	ACTA1 is one of the connected proteins with Annexin 8, however there is no specific role in ovarian cancer and the analysis of protein ATLAS does not show a correlation with survival.	Gou, R., Zhu, L., Zheng, M. *et al.* Annexin A8 can serve as potential prognostic biomarker and therapeutic target for ovarian cancer: based on the comprehensive analysis of Annexins. *J Transl Med* **17,** 275 (2019). https://doi.org/10.1186/s12967-019-2023-z
**FOXO3**	Forkhead Box O3, In Rhabdomyosarcoma-Like 1. This gene belongs to the forkhead family of transcription factors which are characterized by a distinct forkhead domain. This gene likely functions as a trigger for apoptosis through expression of	*FOXO3*, which encodes the transcription factor forkhead box O-3 (FoxO3), is a member of the FOXO subfamily of the forkhead box (FOX) family. FOXO3 can be negatively regulated by	Dall'Acqua, A., Sonego, M., Pellizzari, I., Pellarin, I., Canzonieri, V., D'Andrea, S., Benevol, S., Sorio, R., Giorda, G., Califano, D., Bagnoli, M., Militello, L., Mezzanzanica, D., Chiappetta, G., Armenia, J., Belletti, B., Schiappacassi, M., & Baldassarre, G. (2017). CDK6 protects

	genes necessary for cell death. Gene Ontology (GO) annotations related to this gene include *DNA-binding transcription factor activity* and *protein kinase binding.*	its phosphorylation by the PI3K/Akt signaling pathway and ultimately drives apoptosis when activated. It belongs to the AKT signalling pathway.	epithelial ovarian cancer from platinum-induced death via FOXO3 regulation. *EMBO molecular medicine*, *9*(10), 1415–1433. https://doi.org/10.15252/emmm.201607012
**APOA1**	This gene encodes apolipoprotein A-I, which is the major protein component of high-density lipoprotein (HDL) in plasma.	Apolipoprotein A1 (ApoA1) is remarkably decreased in serum and ovarian tissues of ovarian cancer patients.	Marinho AT, Lu H, Pereira SA, Monteiro E, Gabra H, Recchi C. Anti-tumorigenic and Platinum-Sensitizing Effects of Apolipoprotein A1 and Apolipoprotein A1 Mimetic Peptides in Ovarian Cancer. Front Pharmacol. 2019 Jan 28;9:1524. doi: 10.3389/fphar.2018.01524.

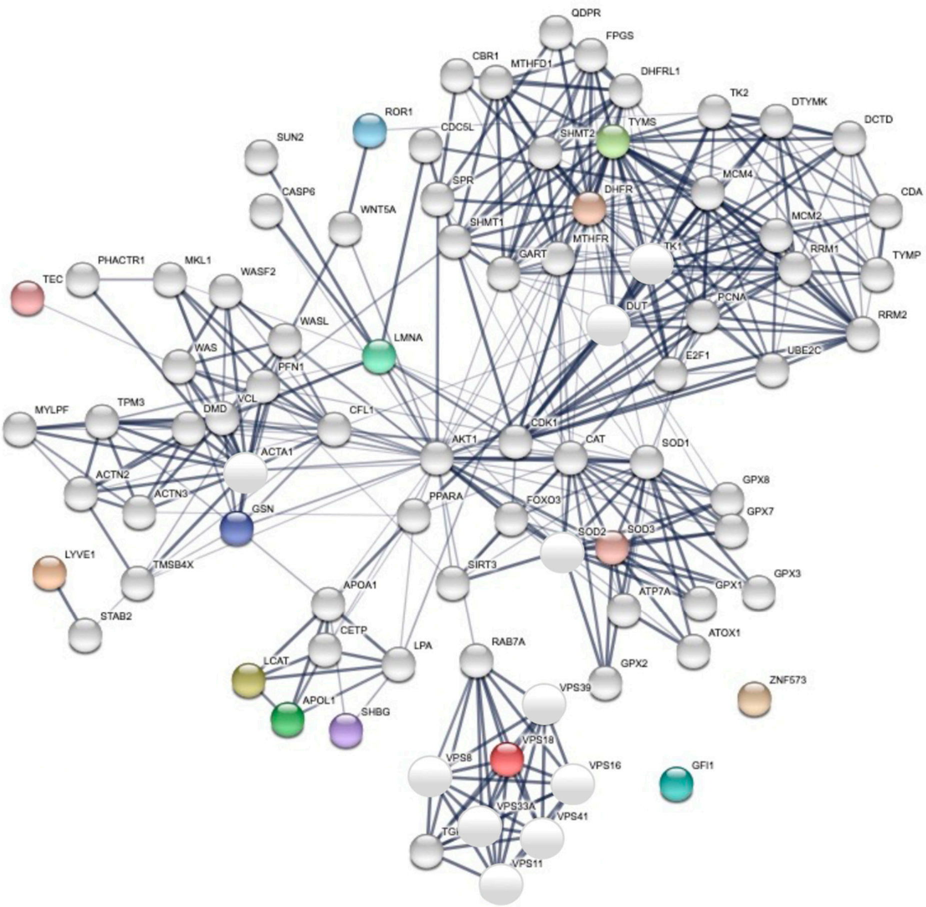

Figure S1. Global network visualization based on STRING pathway enrichment analysis of the 14 selected proteins showing the highest scored biological process. Details are reported in the main text. The 12 selected proteins plus TS and DHFR, were processed using STRING with their annotation to highlight any common biological processes in which they are involved and to identify their interconnections. The colored sphere corresponds to the 14-input protein (12 from MS experiments and TYMS+DHFR) of the selected panel. STRING setting: 1st shell max 10 interactions, 2nd shell max 50 interactions; number of nodes (or proteins): 84; number of edges: 409; average node degree: 9.86; avg. local clustering coefficient: 0.663: expected number of edges: 174; PPI enrichment p-value < $1.0x10^{-16}$).
